# Supplementary material for: APOBEC affects tumor evolution and age at onset of lung cancer in smokers
Source: Nat Commun. 2025 May 21;16:4711. doi: 10.1038/s41467-025-59923-8 (PMC12092836; doi:10.1038/s41467-025-59923-8)
Supplement: Supplementary file 1 — Supplementary Information [file 41467_2025_59923_MOESM1_ESM.pdf]

APOBEC affects tumor evolution and age at onset of lung cancer in smokers

Supplementary Figures

Supplementary Fig. 1

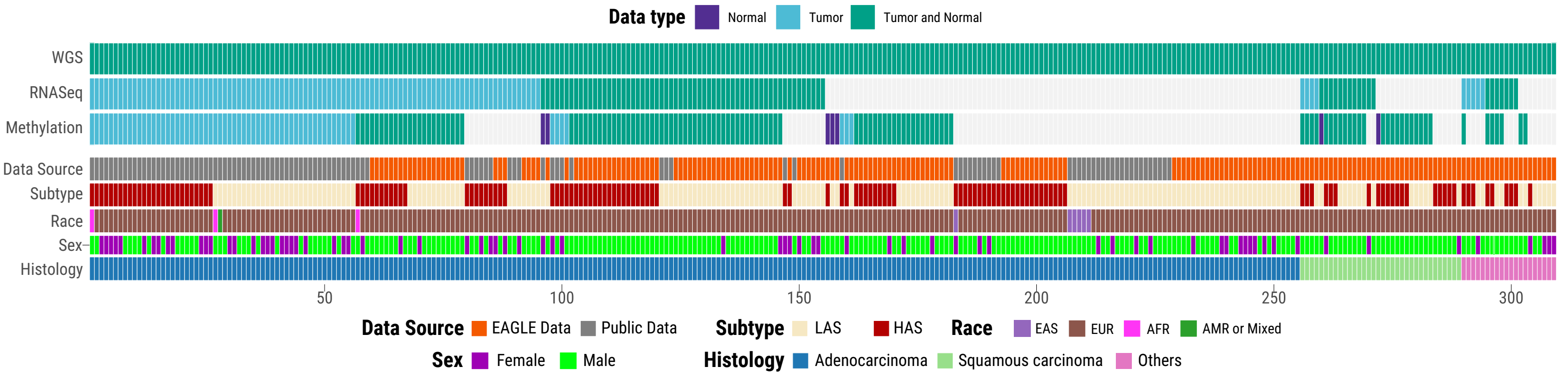

Supplementary Fig. 1: Summary of multi-omics data and clinical information in this study.

Supplementary Fig. 2

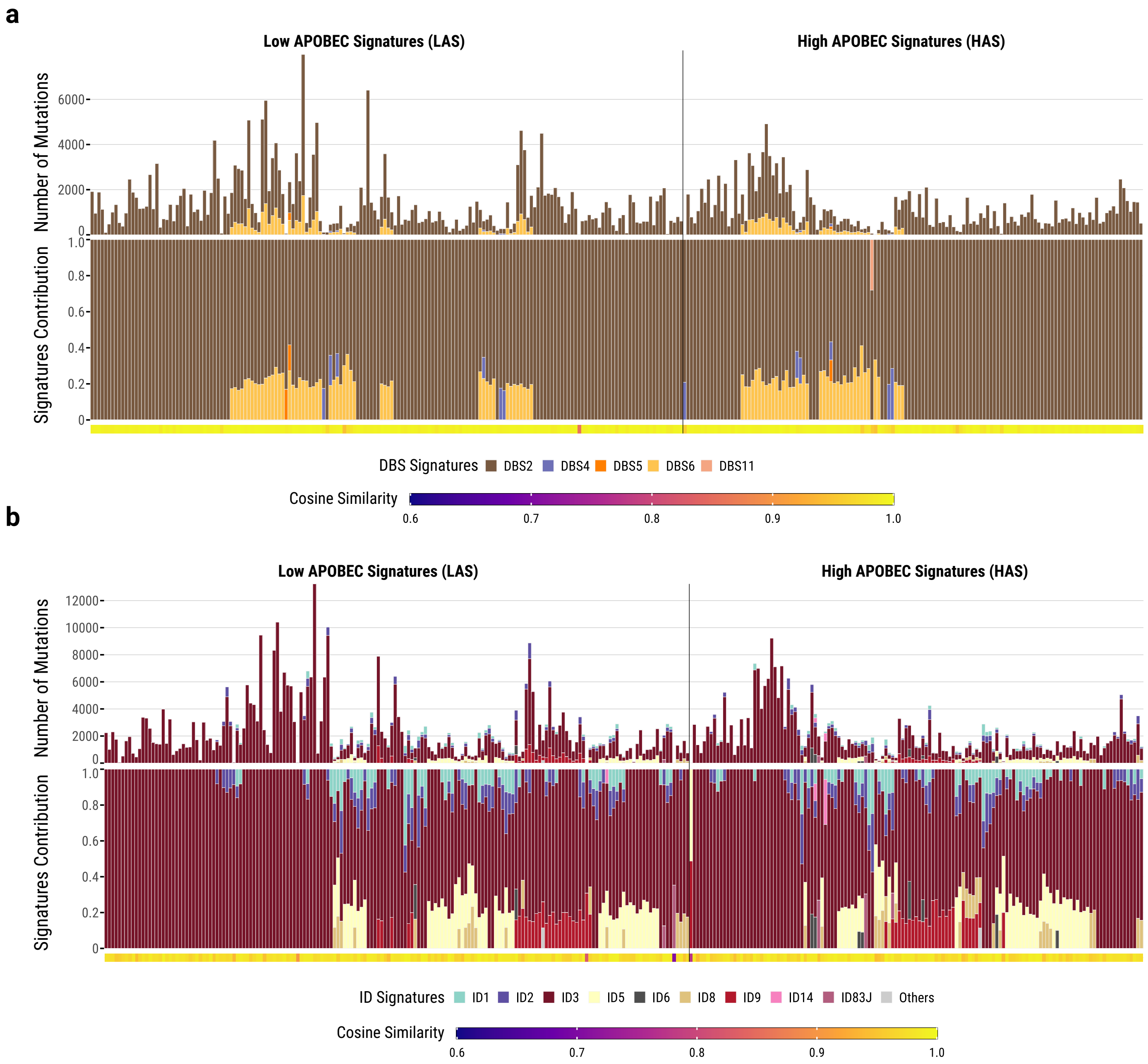

Supplementary Fig. 2: Landscape of mutational processes for DBS (a) and ID (b). The landscape of mutational signature plots include a bar plot presenting the total number of mutations assigned to each signature, the proportion plot of signatures assigned to each sample, and cosine similarity between the original mutation profile and signature decomposition.

Supplementary Fig. 3

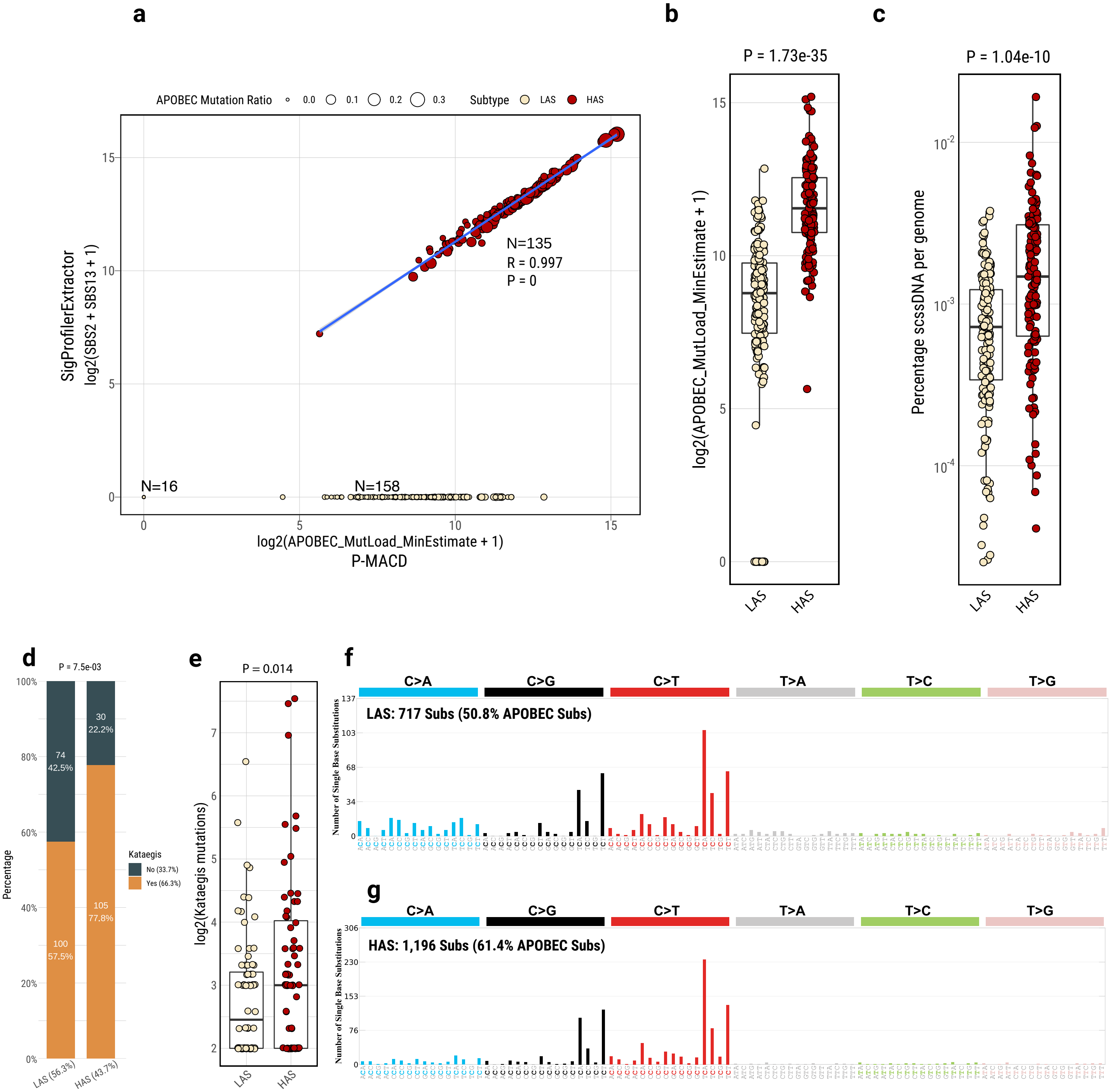

Supplementary Fig. 3: Characterization of APOBEC mutagenesis based on the P-MACD algorithm and *kataegis* between LAS and HAS tumors. a, Comparison of APOBEC mutational signatures detection between the NMF-based SigProfileExtractor approach and the mutational pattern-based P-MACD approach. APOBEC mutation detection is almost identical between the two approaches in 135 tumors, while P-MACD identifies APOBEC mutations below the detection limit of SigProfileExtractor in 158 tumors. **b,c,** Comparisons of minimal estimated APOBEC mutation load (**b**) and percentage of hypermutable strand-coordinated ssDNA (scssDNA) per genome due to A3A mutagenesis (**c**) between LAS and HAS tumors. The P-values derived from the two-sided Wilcoxon sum rank test are shown above the plots. **d,** Comparisons of *kataegis* frequency between LAS and HAS tumors. P-values of the two sided Fisher's exact test are shown on the top of the barplot. **e,** Number of mutations in *kataegis* between LAS and HAS tumors. The P-values derived from the two-sided Wilcoxon sum rank test are shown above the plots. **f-g,** Mutational spectrum of total mutations contributing to *kataegis* in LAS (**f**) and HAS (**g**) tumors, respectively. All box plots display the median (centerline), interquartile range (box), and whiskers extending to 1.5× the interquartile range (IQR) by default in ggplot2.

Supplementary Fig. 4

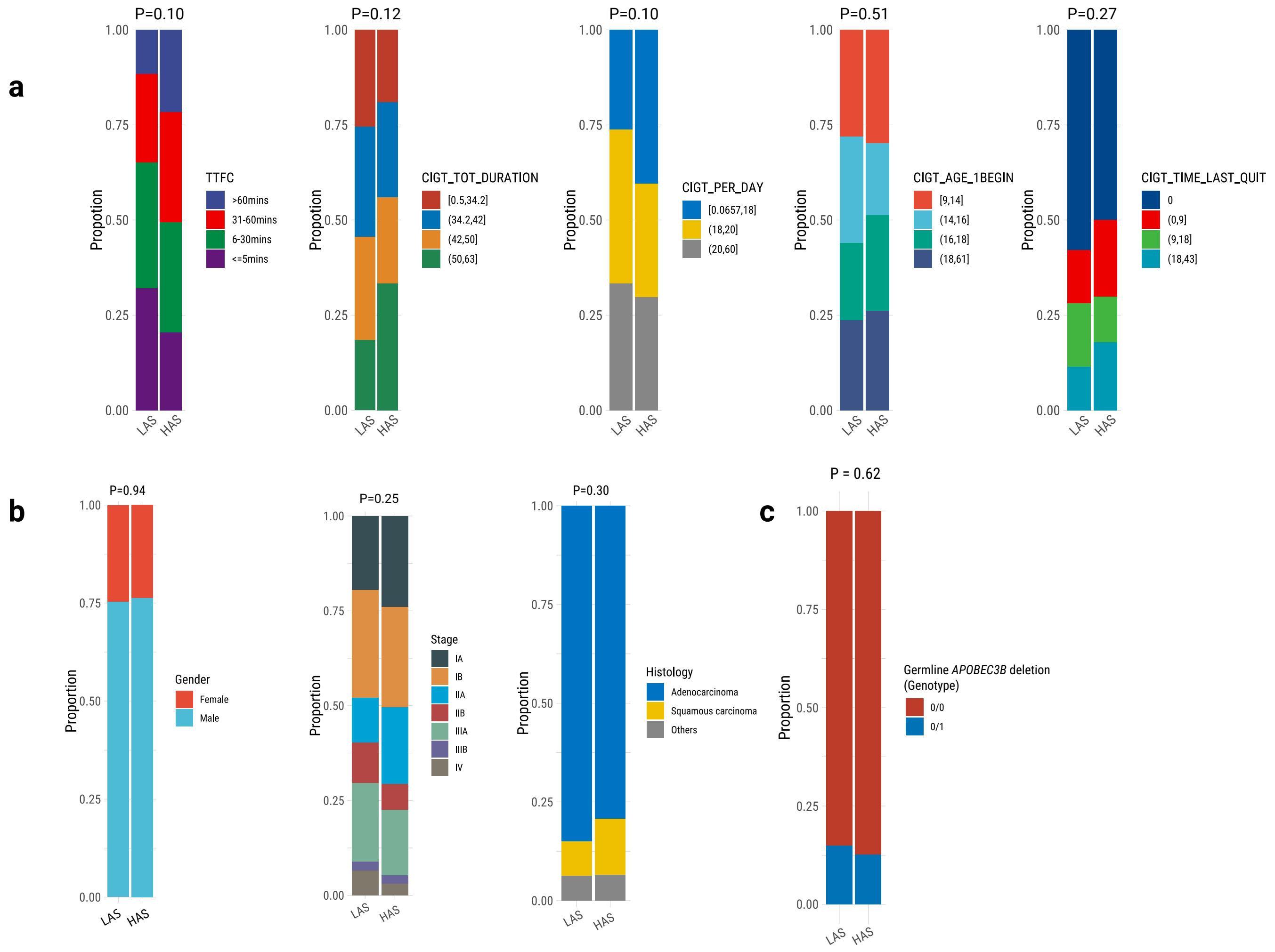

Supplementary Fig. 4: a-c, Comparison of tobacco smoking exposure (a), clinical information (b), and germline APOBEC3B deletion (c) between LAS and HAS tumors. The P-values derived from the two-sided Chi-squared test are shown above the plots.

Supplementary Fig. 5

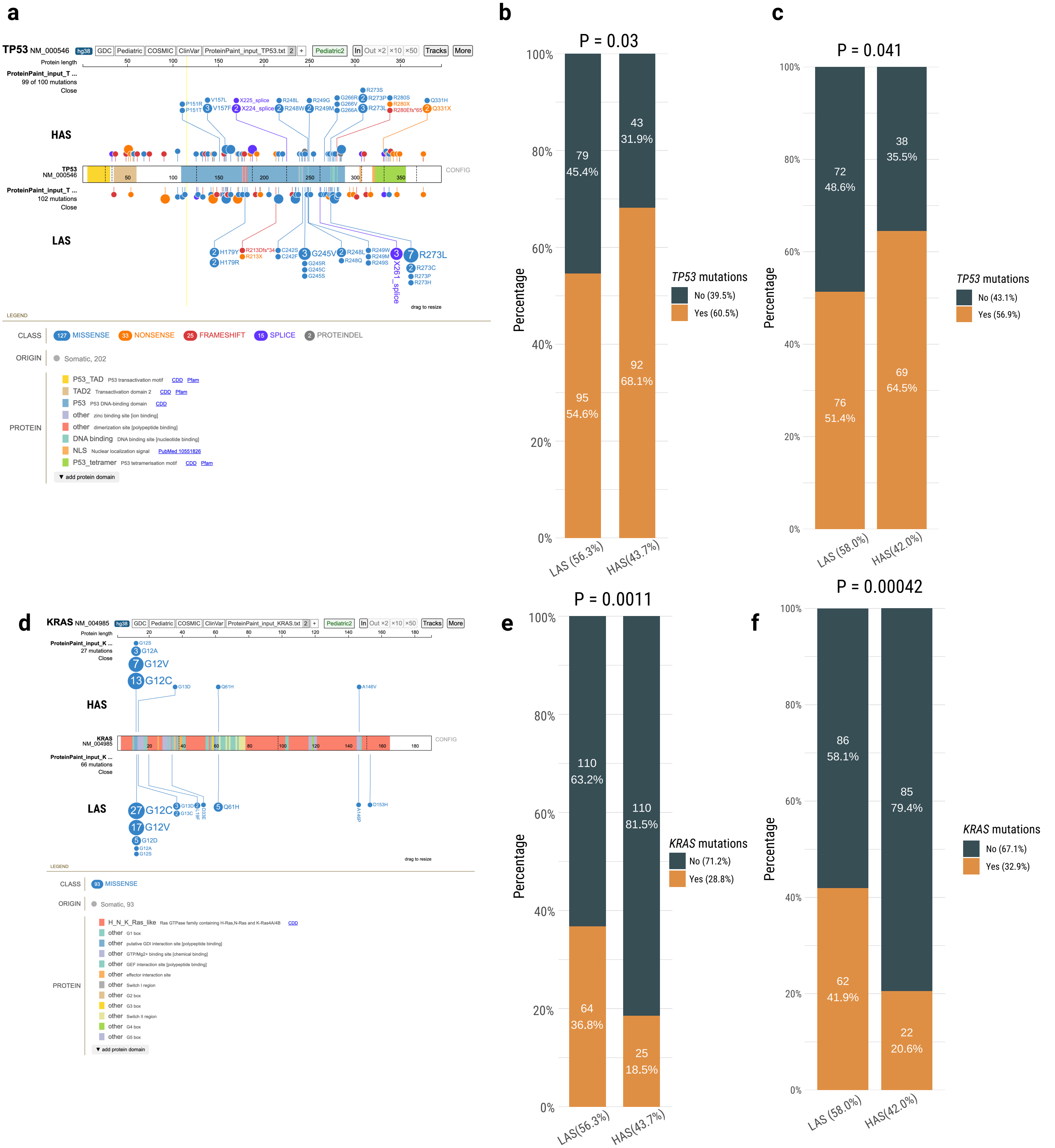

Supplementary Fig. 5: Lollipop plot and enrichment analysis of *TP53* and *KRAS* mutations in LAS and HAS tumors. a, Lollipop plot illustrating *TP53* mutations across all samples. **b-c**, Enrichment analysis of *TP53* mutations comparing LAS and HAS tumors in (**b**) all samples and (**c**) LUAD samples only. **d**, Lollipop plot illustrating *KRAS* mutations across all samples. **e-f**, Enrichment analysis of *KRAS* mutations comparing LAS and HAS tumors in (**e**) all samples and (**f**) LUAD samples only. P-values of the two sided Fisher's exact test are shown on the top of each barplot.

Supplementary Fig. 6

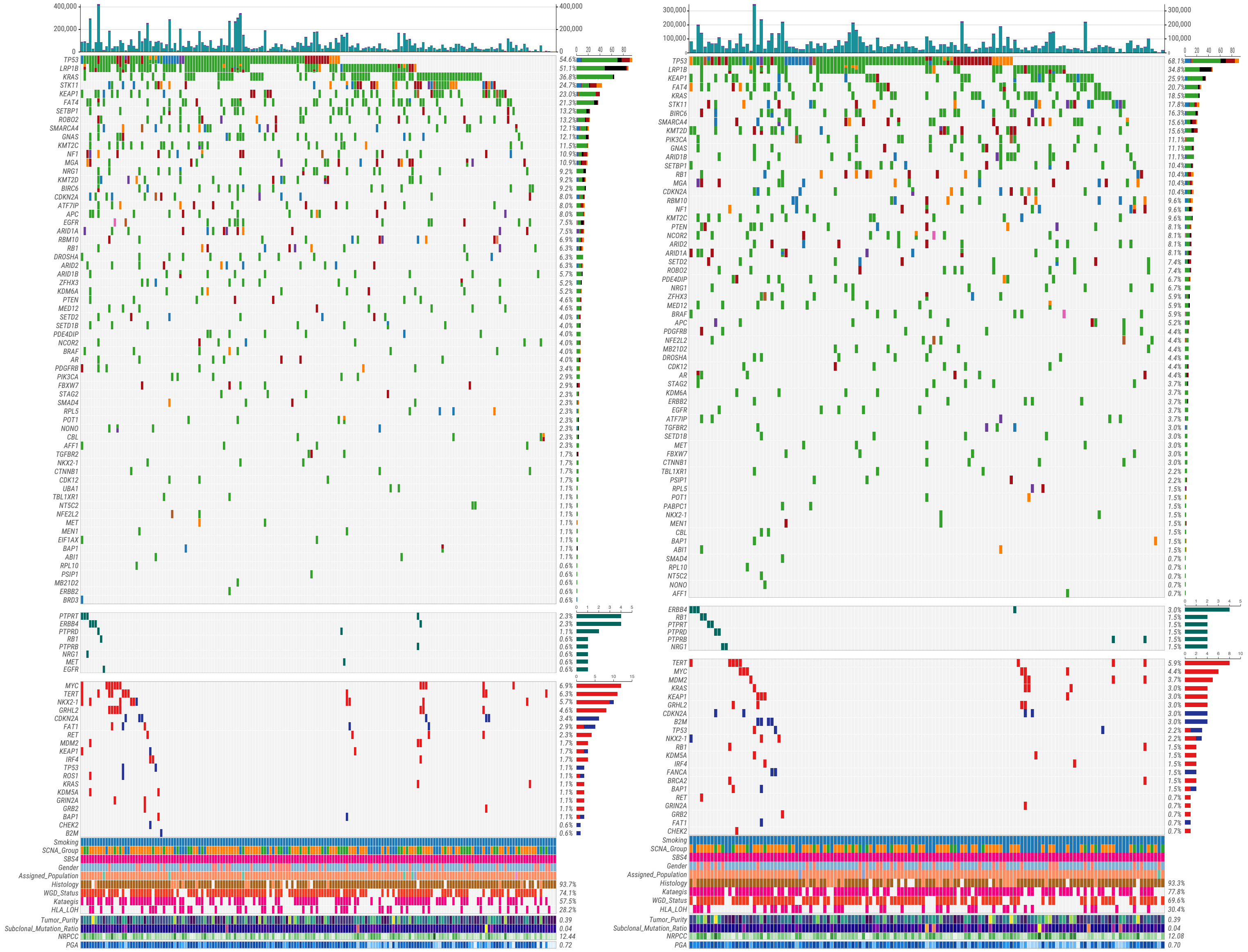

LAS HAS

Supplementary Fig. 6: Genomic characterizations of LAS and HAS tumors. The subplots from top to bottom: distribution of genomic alteration numbers, most frequently mutated or potential driver genes, oncogenic fusions, significant focal SCNA, and different genomic features. The numbers on the right of each subplot show the overall frequency or median values.

Supplementary Fig. 7

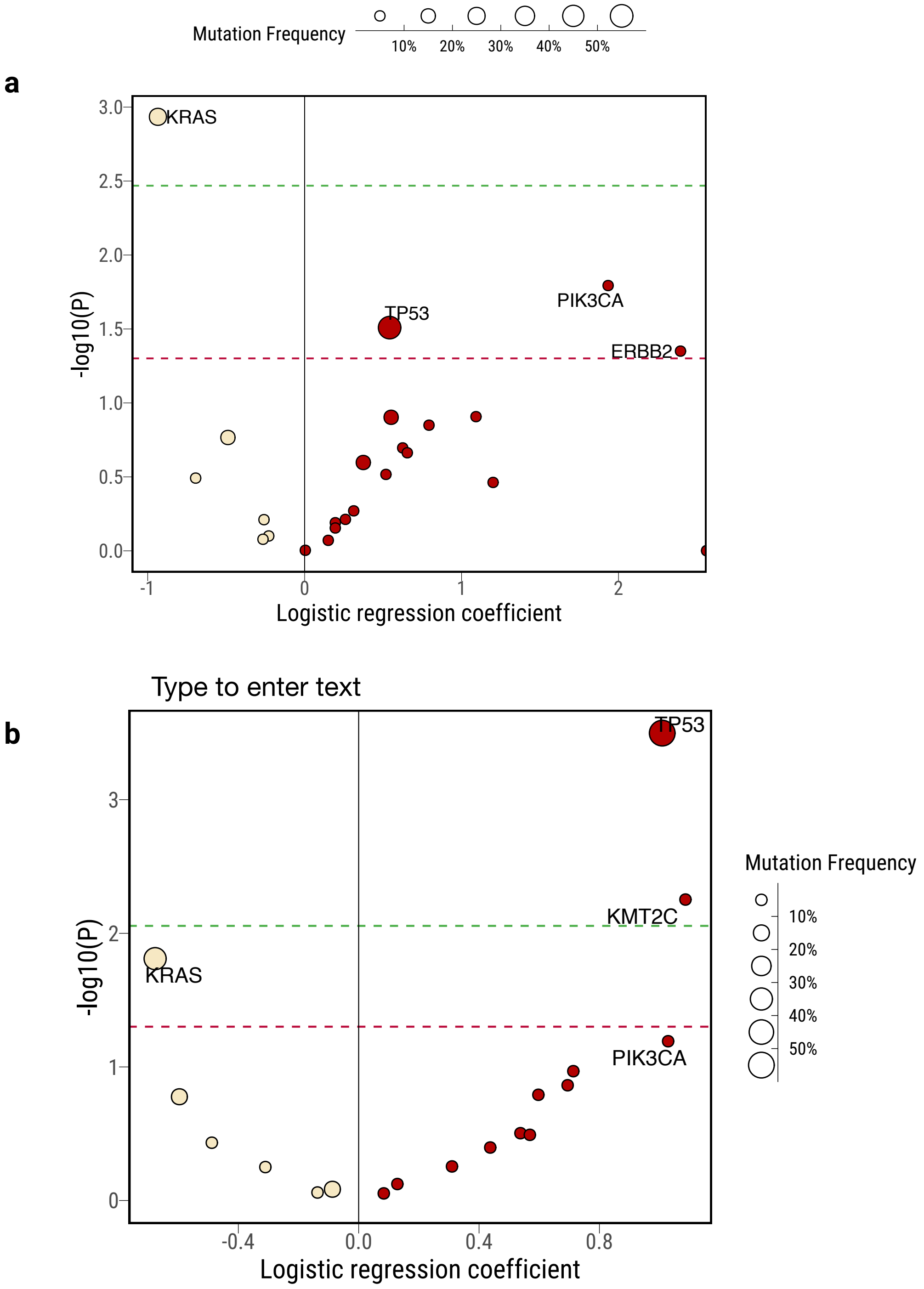

Supplementary Fig. 7: a,b, Logistic regression analysis between tumor subtypes and driver mutation status of driver genes in this study (a) and TCGA LUAD study (b), adjusting for the following covariates: age, sex, histology, TMB, and tumor purity. Driver mutations were identified based on the Cancer Genome Interpreter platform. The significance thresholds P<0.05 (red) and FDR<0.05 (green) are indicated by the dashed lines.

Supplementary Fig. 8

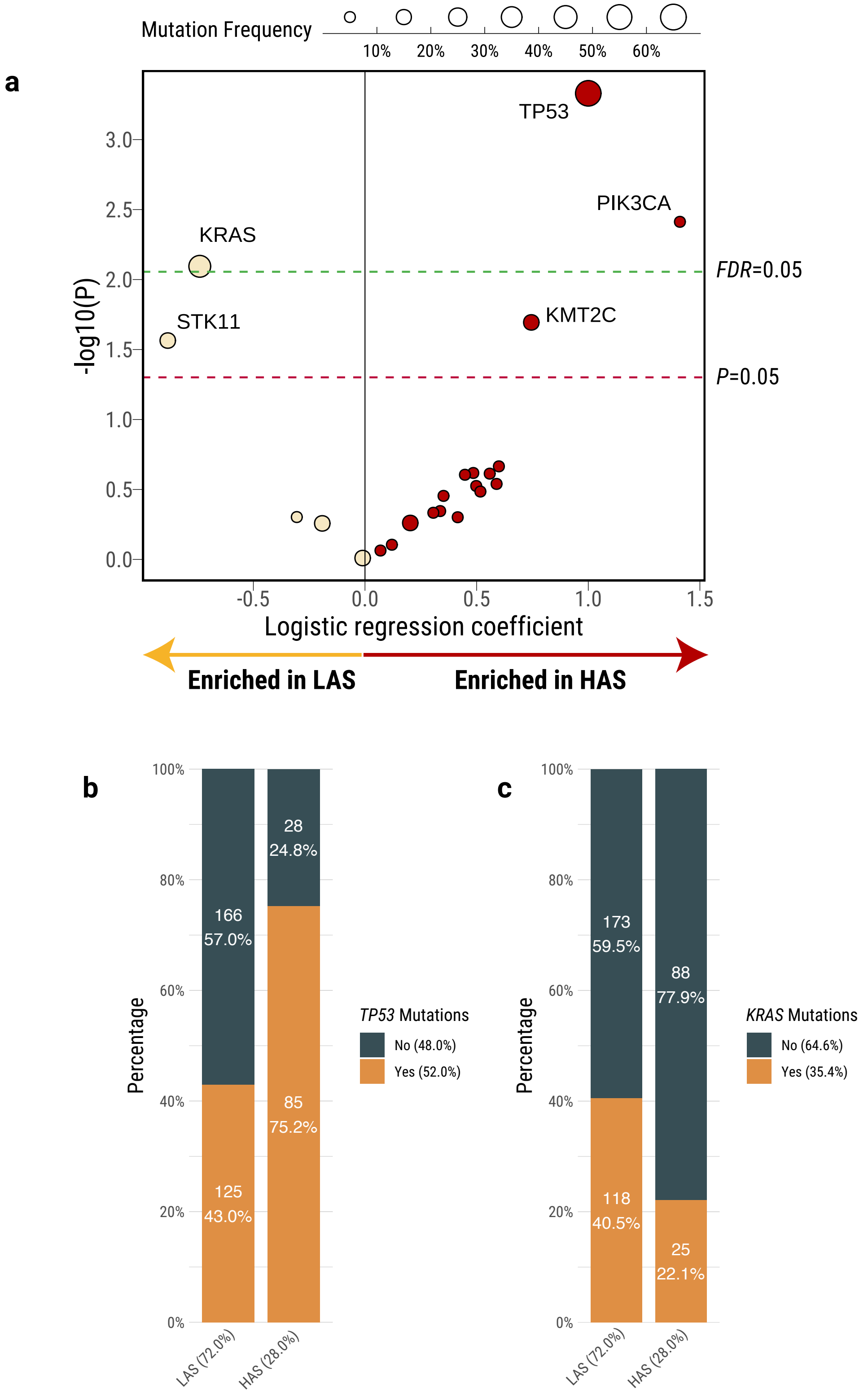

Supplementary Fig. 8: a, Logistic regression analysis between tumor subtypes and nonsynonymous mutation status of driver genes in TCGA LUAD dataset, adjusting for the following covariates: age, sex, histology, TMB, and tumor purity. The significance levels P<0.05 (red) and FDR<0.05 (green) are indicated by the dashed lines. Bar plots show *TP53* (**b**) and *KRAS* (**c**) mutation enrichment between LAS and HAS tumors.

Supplementary Fig. 9

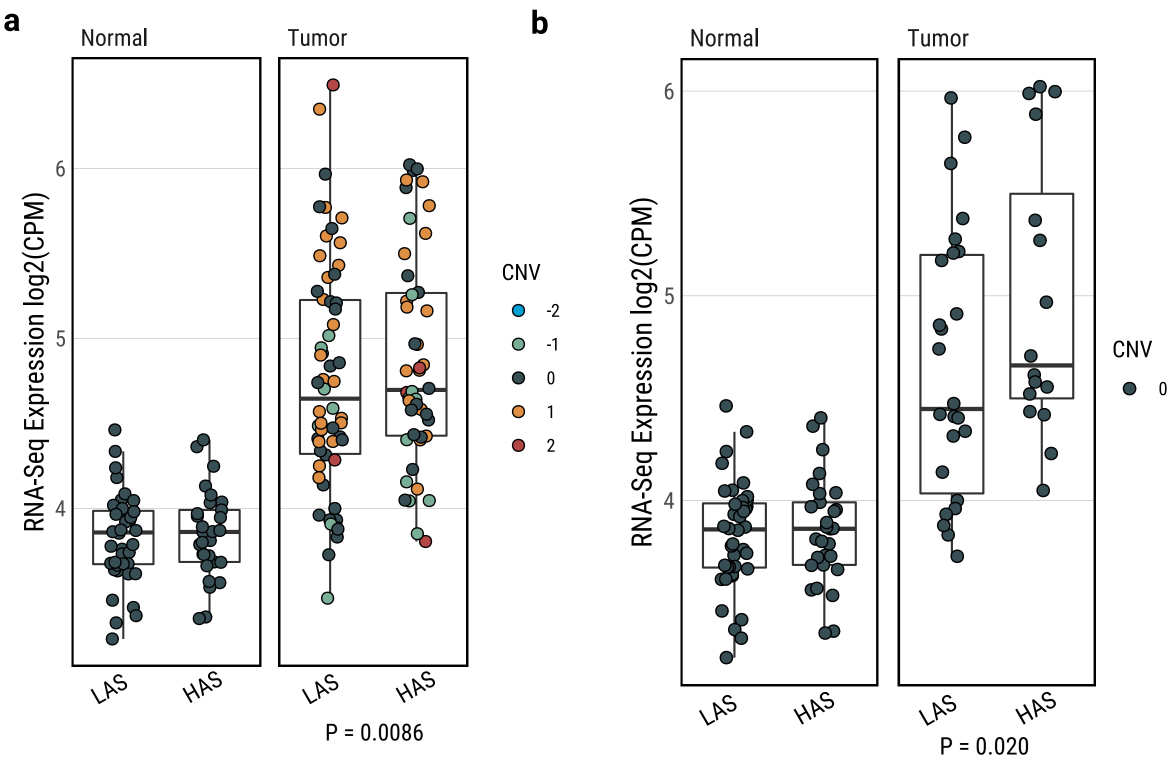

Supplementary Fig. 9: Differentially expressed *UNG* between LAS and HAS tumors based on all available tumors (a) or only in tumors with copy-neutral status for the *UNG* genomic location (b). Significant P-values from the linear regression are labeled below each boxplot. The linear regression model was adjusted for the following covariates: tumor purity and copy number status in (**a**) and tumor purity only in (**b**). All box plots display the median (centerline), interquartile range (box), and whiskers extending to 1.5× the interquartile range (IQR) by default in ggplot2.

Supplementary Fig. 10

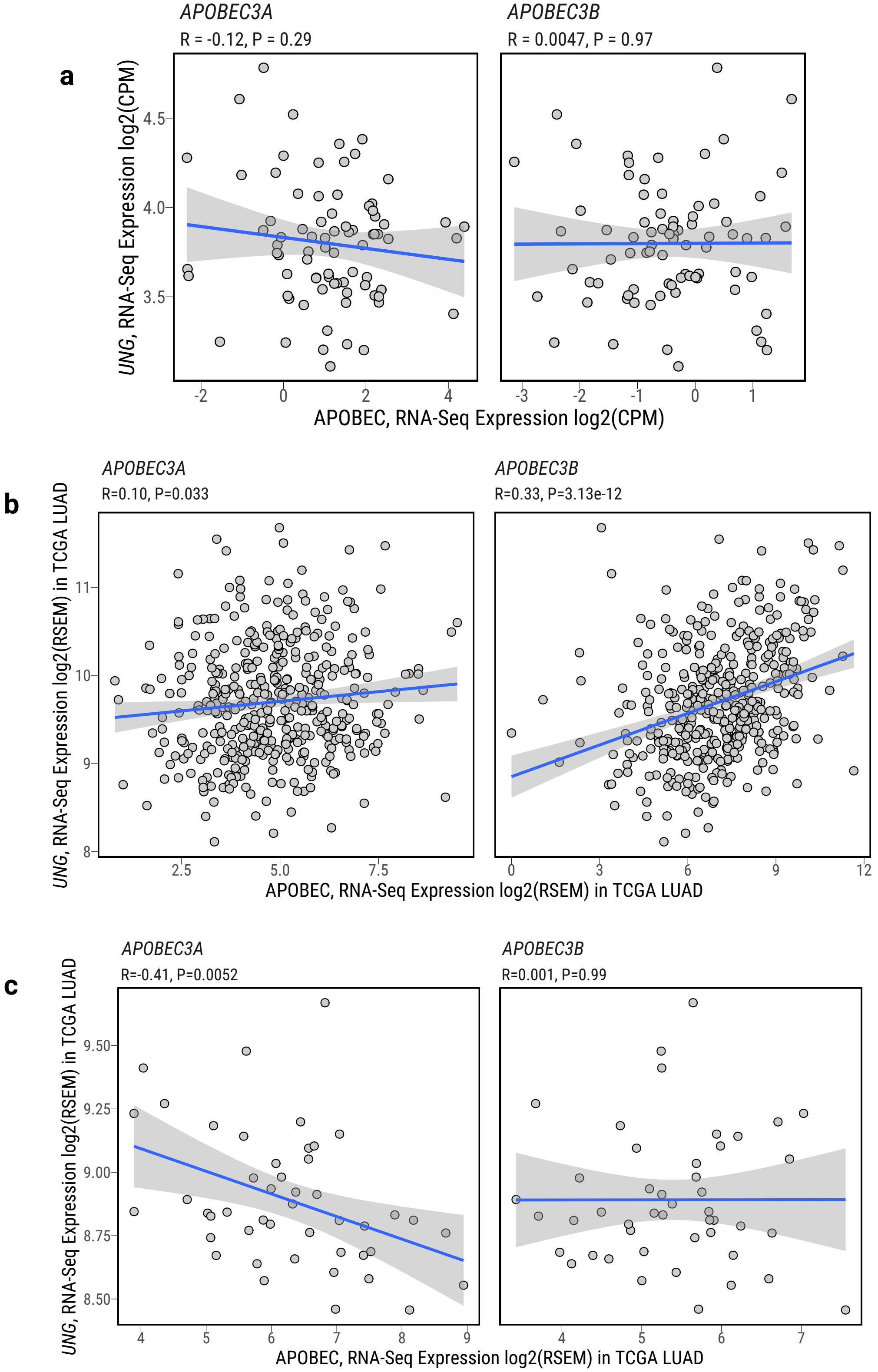

Supplementary Fig. 10: Gene expression correlation between *UNG* and *APOBEC3A* or *APOBEC3B* in normal samples from this study (a), tumor samples from TCGA LUAD (b), and normal samples from TCGA LUAD (c). Significant P-values and Pearson correlation coefficients are shown on the top of each scatter plot. The shaded area represents the 95% confidence level.

Supplementary Fig. 11

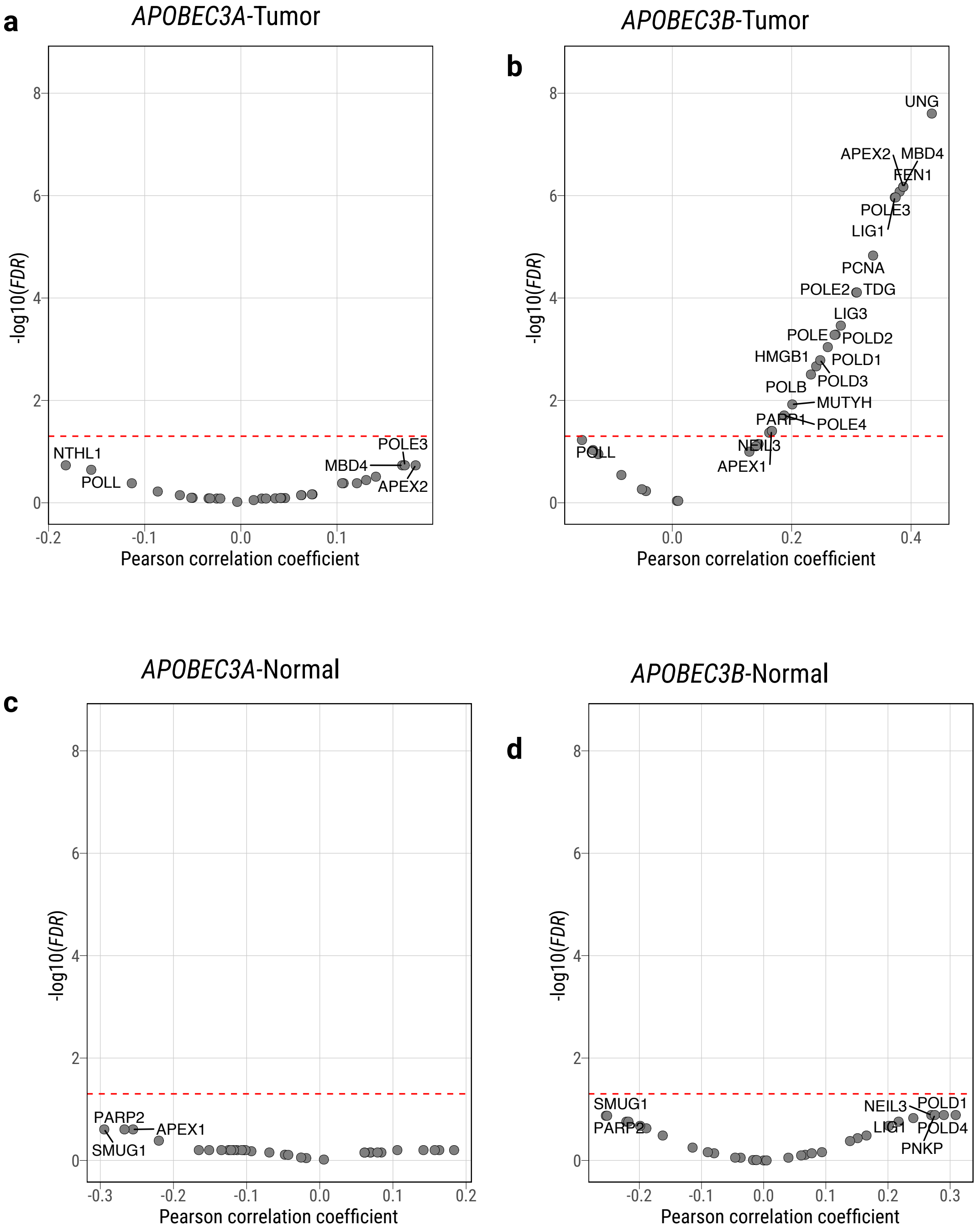

Supplementary Fig. 11: Volcano plots of the expression correlations between genes in the base excision repair pathway and *APOBEC3A* (a) or *APOBEC3B* (b) in tumors, and between genes in the base excision repair pathway and *APOBEC3A* (c) or *APOBEC3B* (d) in normal samples. The red dashed line indicates the significance threshold FDR=0.05. Multiple testing correction was applied using the Benjamini–Hochberg (BH) method.

Supplementary Fig. 12

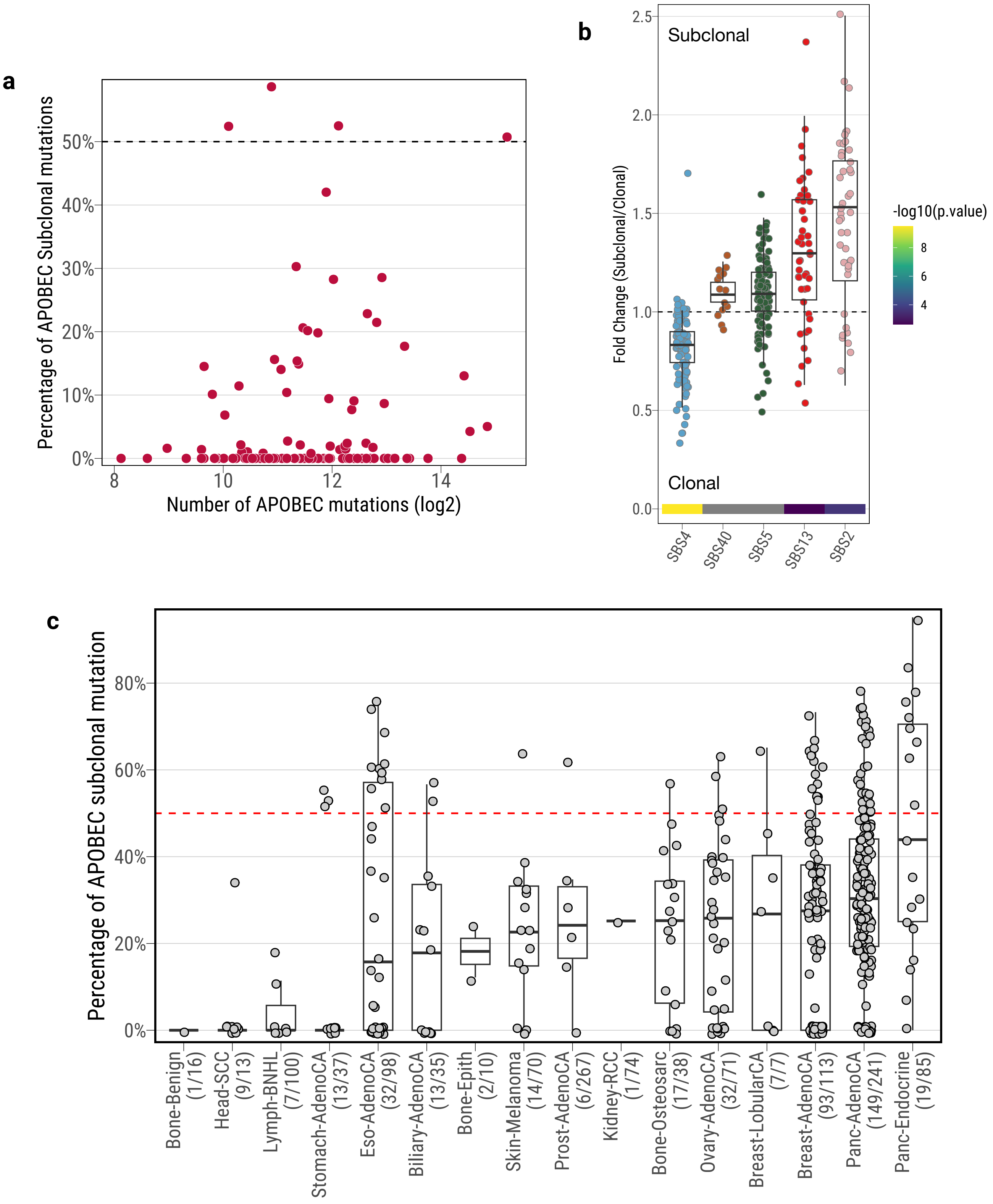

Supplementary Fig. 12: APOBEC mutation clonality. a, Percentage of APOBEC subclonal mutations in each tumor from this study. **b**, Fold changes between relative proportions of subclonal and clonal mutations attributed to individual mutational signatures. The P-values derived from the two-sided Wilcoxon sum rank test are shown on the bottom of the plots. **c**, Percentage of APOBEC subclonal mutations in all cancer types with clonality data from the PCAWG study. For each cancer type, the x-axis shows the number of samples with APOBEC mutations over the total number of samples with clonality data. All box plots demarcate the first and third quartiles of the distribution, with the median shown in the center and whiskers covering data within 1.5× the IQR from the box.

Supplementary Fig. 13

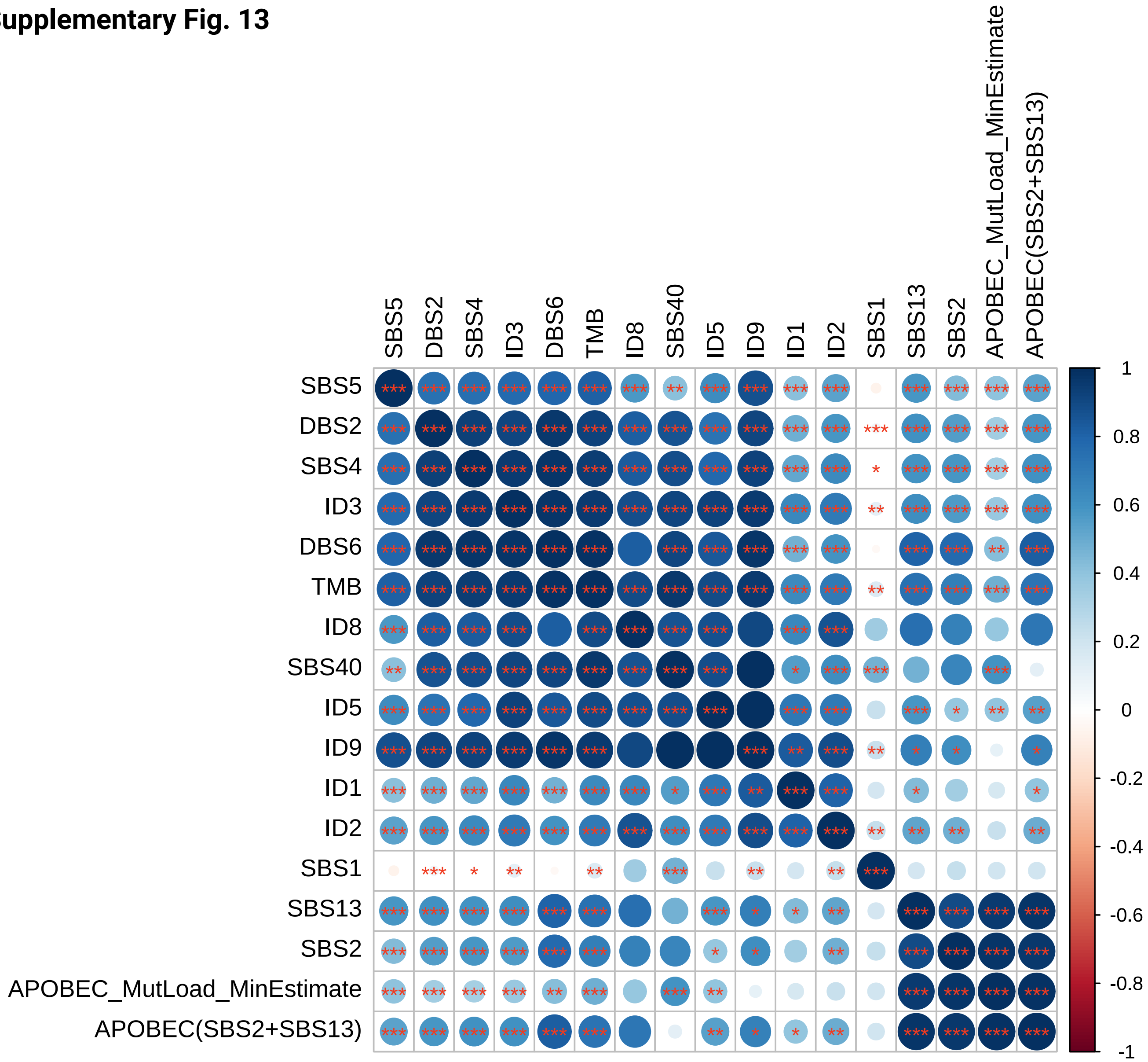

Supplementary Fig. 13: Pairwise Pearson correlation between the number of mutations assigned to two observed mutational signatures or overall TMB. The color and size of the point indicate the correlation coefficients. *P<0.05, ∗∗P<0.01 and ∗∗∗P<0.001.

Supplementary Fig. 14

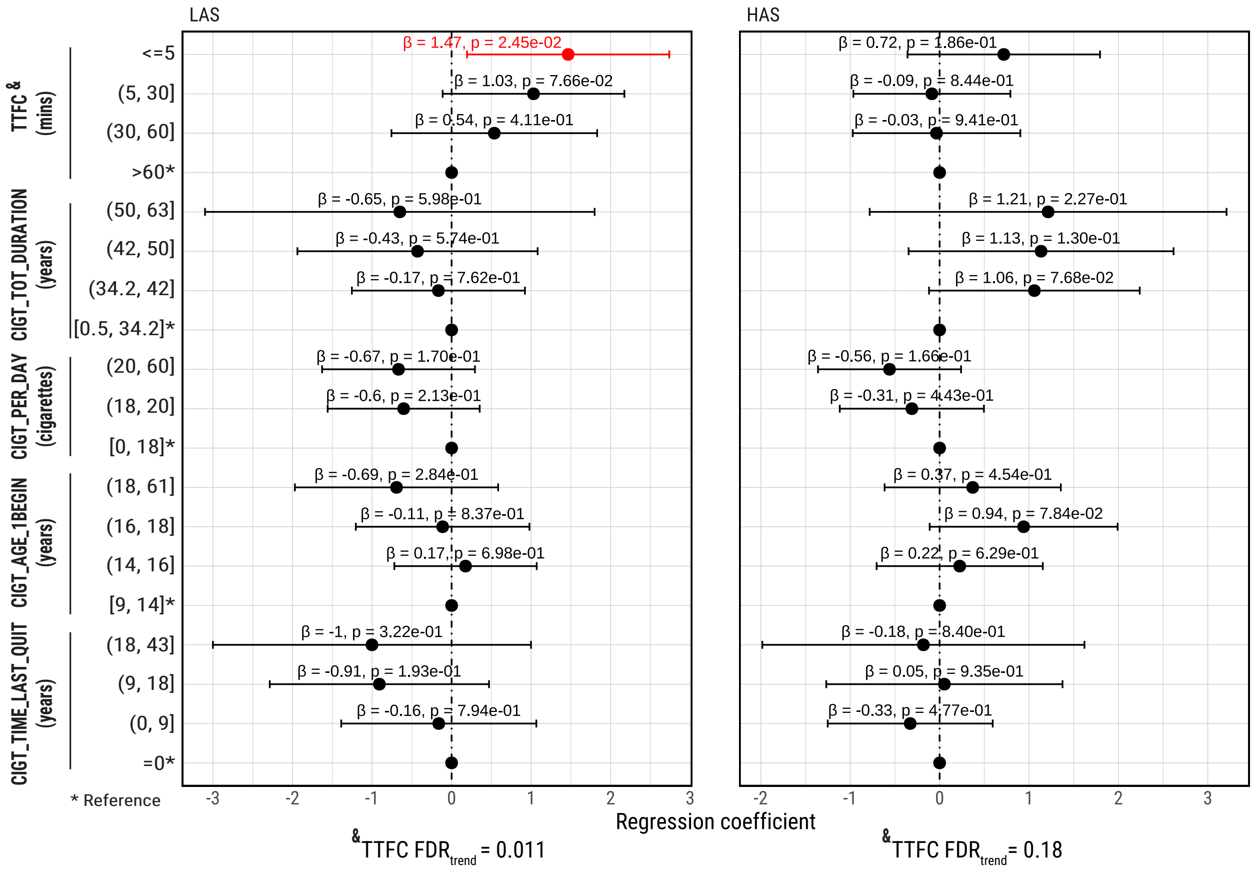

Supplementary Fig. 14: Forest plot for the associations between TMB and smoking variables, stratified between LAS and HAS tumors in LUAD samples only. Forest plot for the associations between TMB and smoking variables, stratified between LAS and HAS tumors. P-values and regression coefficients with 95% confidence intervals (CIs) are shown for each category of smoking variables. Significant associations are in red ink. Trend test *P*-values, adjusted for multiple testing using the Benjamini–Hochberg method (FDR_{trend}) from associations between TTFC and TMB are included below the forest plots.

Supplementary Fig. 15

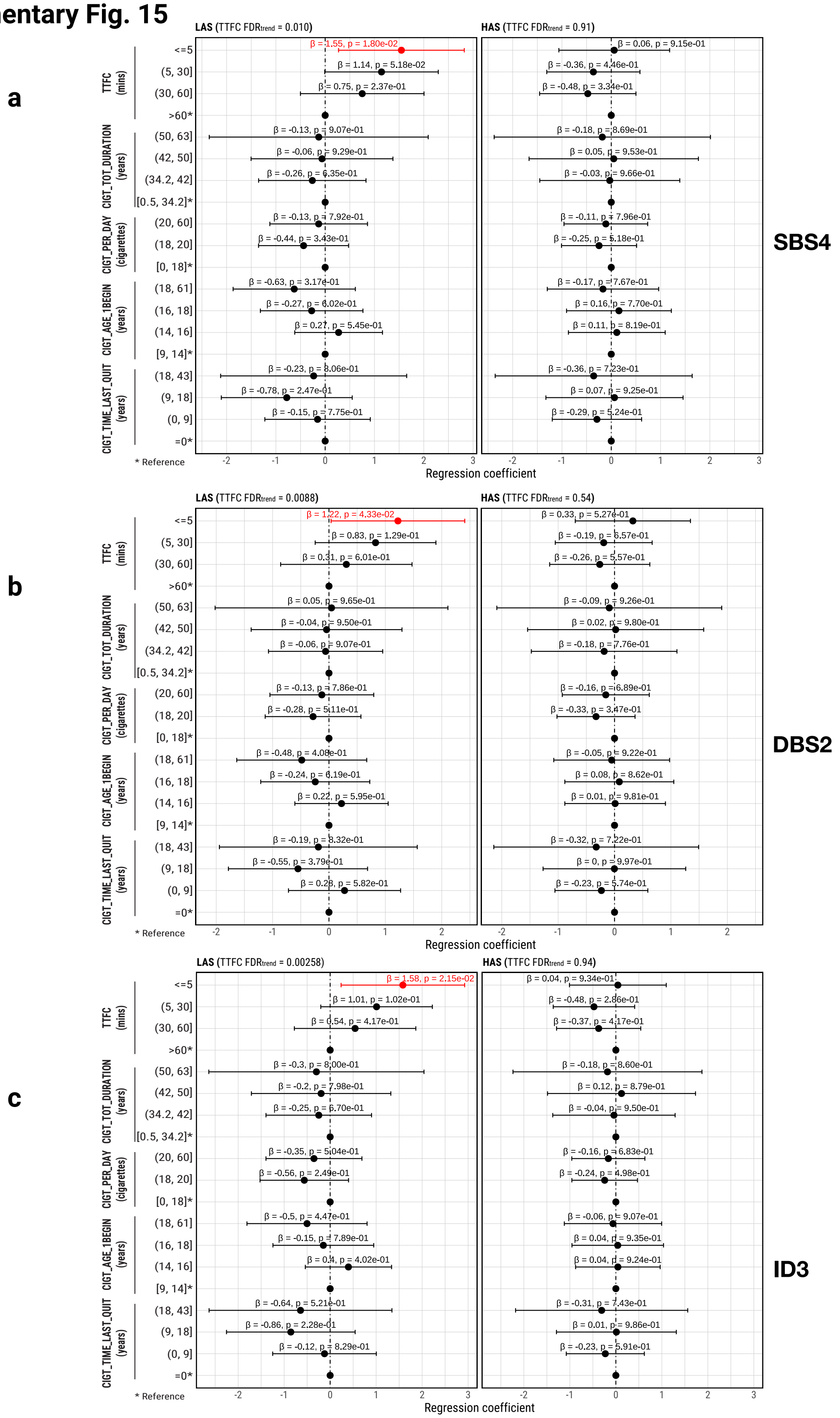

Supplementary Fig. 15: Multivariate regression analysis between five smoking variables and the number of mutations assigned to smoking-associated signatures including SBS4 (a), DBS2 (b), and ID3 (c) in EAGLE samples (n=198). Linear regression analyses performed between LAS and HAS tumors. P-values and regression coefficients with 95% CIs from forest plots are shown for each category of smoking variables. Significant associations are in red ink. Trend test P-values (P_{trend}) from associations of TTFC with SBS4, DBS2, and ID3 are included below the forest plots. All association analyses are adjusted for the following covariates: age, sex, histology, and tumor purity. Trend test *P*-values, adjusted for multiple testing using the Benjamini–Hochberg method (FDR_{trend}) from associations between TTFC and TMB are included above the forest plots.

Supplementary Fig. 16

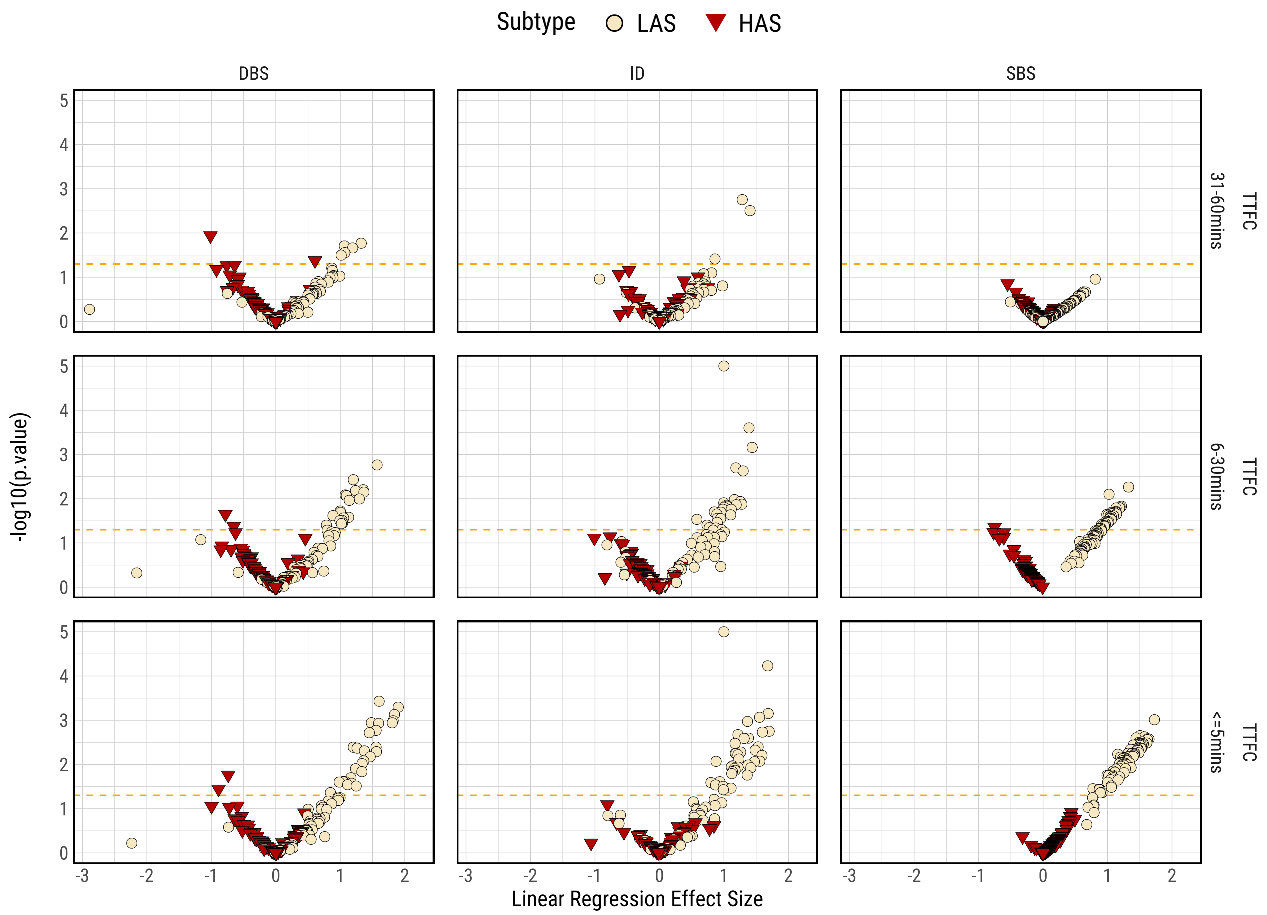

Supplementary Fig. 16: Linear regression between the smoking variable TTFC and the number of mutations among SBS, DBS, and ID in LAS and HAS tumors (n=198). TTFC >60 mins is used as the reference for the associations, adjusting for the following covariates: age, sex, histology, and tumor purity. The associations are performed by separating the LAS (circles) and HAS (triangles) tumors. The orange dashed line indicates the significance threshold of P=0.05.

Supplementary Fig. 17

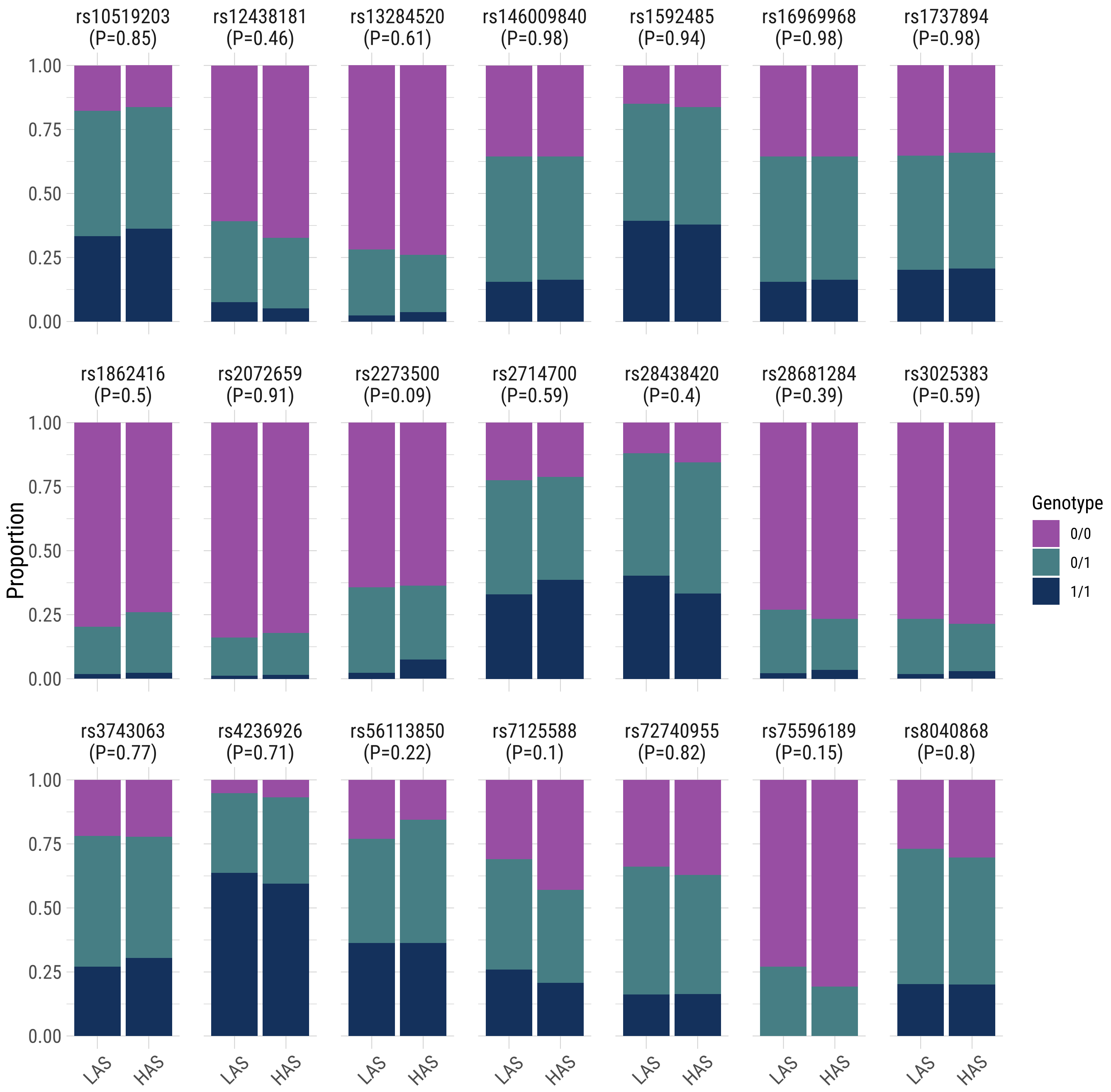

Supplementary Fig. 17: Comparison of germline variants from a GWAS of nicotine addiction between LAS and HAS tumors. The P-values derived from the two-sided Chi-squared test are shown above the plots.

Supplementary Fig. 18

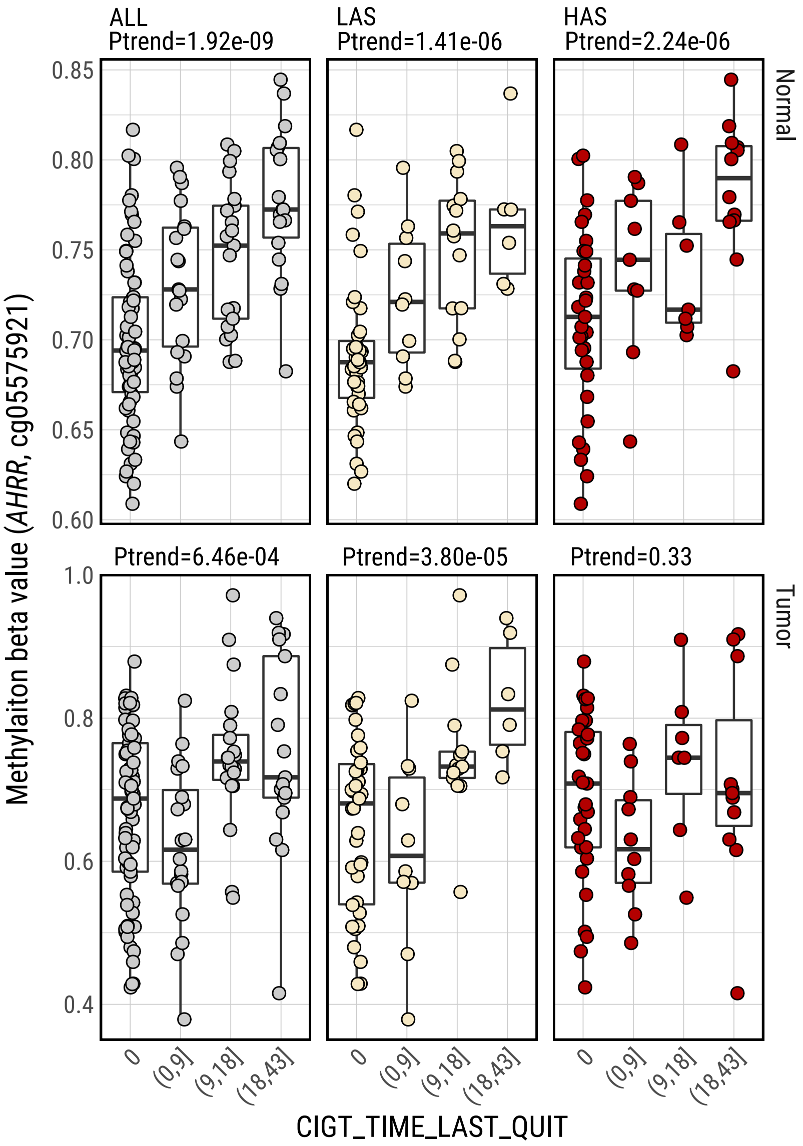

Supplementary Fig. 18: **Multivariate regression analysis between the DNA methylation level of CpG probe cg05575921 within *AHRR* and the smoking variable CIGT_TIME_LAST_QUIT [Number of years since the subject quitted smoking cigarettes (0 means current smokers)] in tumor and normal EAGLE tissue samples (n=122).** The association analyses are performed on all tumors and separately between LAS and HAS tumor subtypes. Trend test P-values (P_{trend}) are labeled above each subplot. The linear regression model was adjusted for the following covariates in tumor: age, sex, histology, and tumor purity; and in normal tissue: age and sex. All box plots display the median (centerline), interquartile range (box), and whiskers extending to 1.5× the interquartile range (IQR) by default in ggplot2.

Supplementary Fig. 19

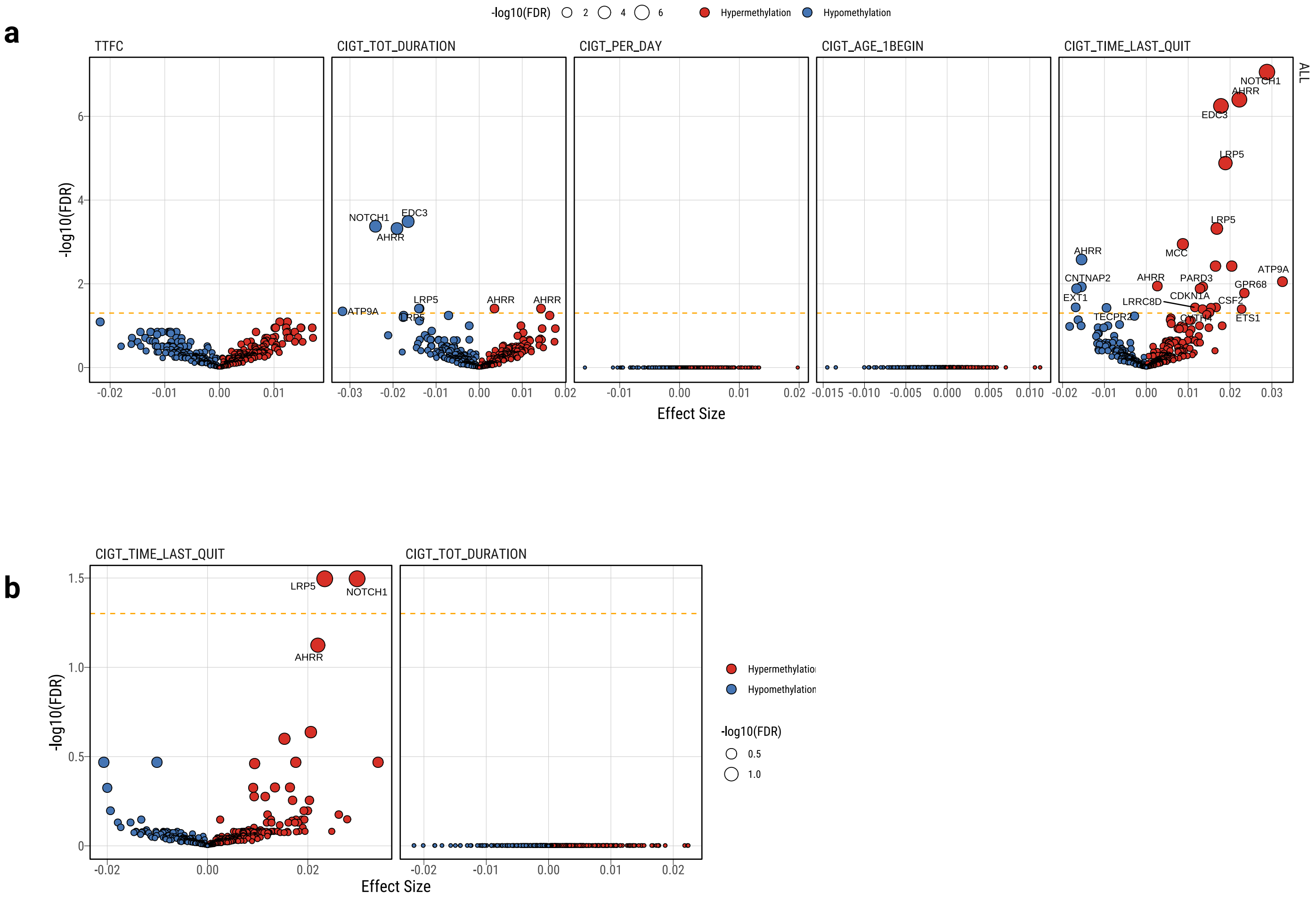

Supplementary Fig. 19: Linear regression analysis between five smoking variables and DNA methylation levels of selected CpG probes in normal EAGLE samples (n=122). a,b, Volcano plots of the associations between methylation levels of each CpG probe and smoking variables independently (**a**) or with CIGT_TIME_LAST_QUIT [Number of years since the subject quitted smoking cigarettes (0 means current smokers)] and CIGT_TOT_DURATION (Total period (in years) during which the subject smoked cigarettes regularly. (**b**) Association FDR values (adjusted using the Benjamini-Hochberg method) are shown on the y-axis of each volcano plot. The orange dashed line indicates the associations with FDR<0.1. The CpG probes associated with tobacco smoking are derived from a previous study comparing methylation levels between smokers and never smokers in normal lung tissue. The size and color of each point represent the FDR and association direction, respectively. All association analyses are adjusted for the following covariates: age and sex.

Supplementary Fig. 20

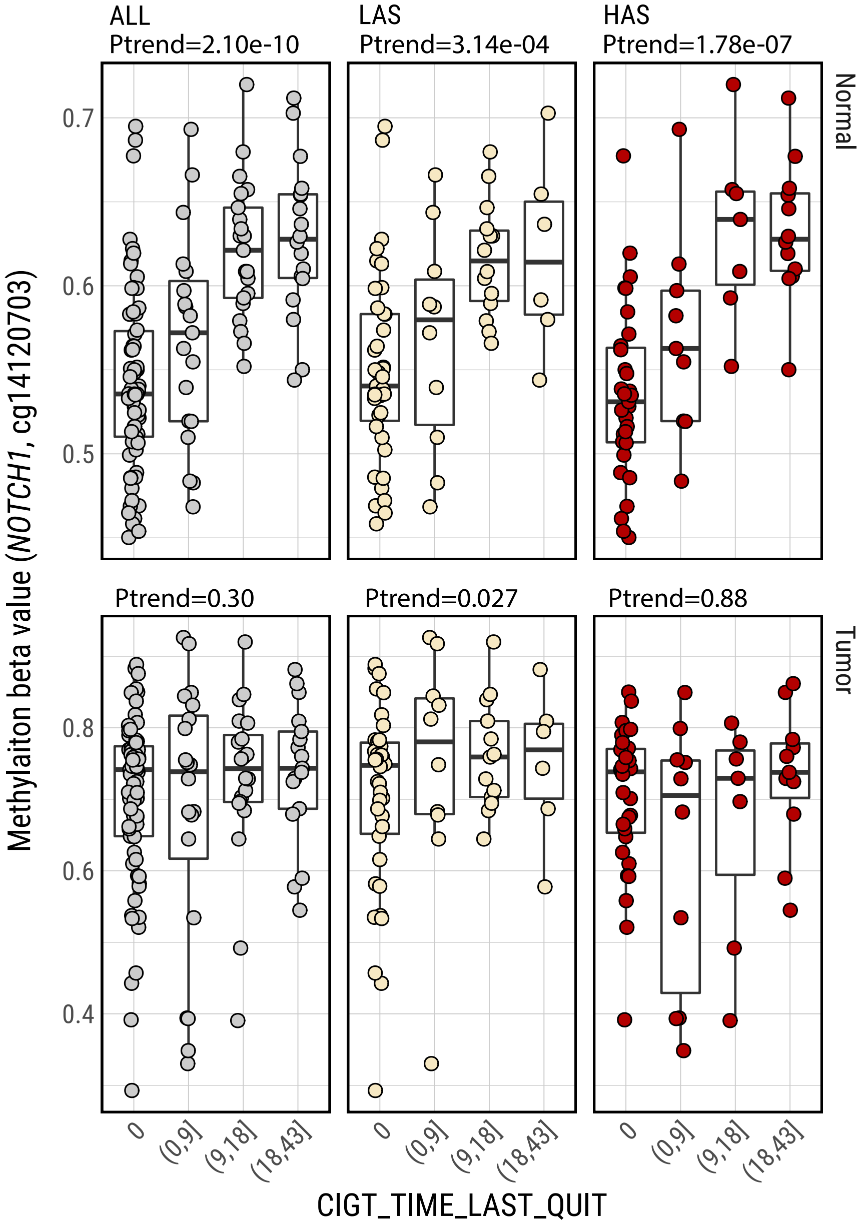

Supplementary Fig. 20: **Multivariate regression analysis between the smoking variable CIGT_TIME_LAST_QUIT [Number of years since the subject quitted smoking cigarettes (0 means current smokers)] and DNA methylation level of CpG probe cg14120703 within *NOTCH1* in tumor or normal EAGLE tissue samples (n=122).** The association analyses are performed on all subjects and separately between LAS and HAS tumor. Trend test P-values (P_{trend}) are labeled above each subplot. The linear regression model was adjusted for the following covariates in tumor: age, sex, histology, and tumor purity; and in normal tissue: age and sex. All box plots display the median (centerline), interquartile range (box), and whiskers extending to 1.5× the interquartile range (IQR) by default in ggplot2.

Supplementary Fig. 21

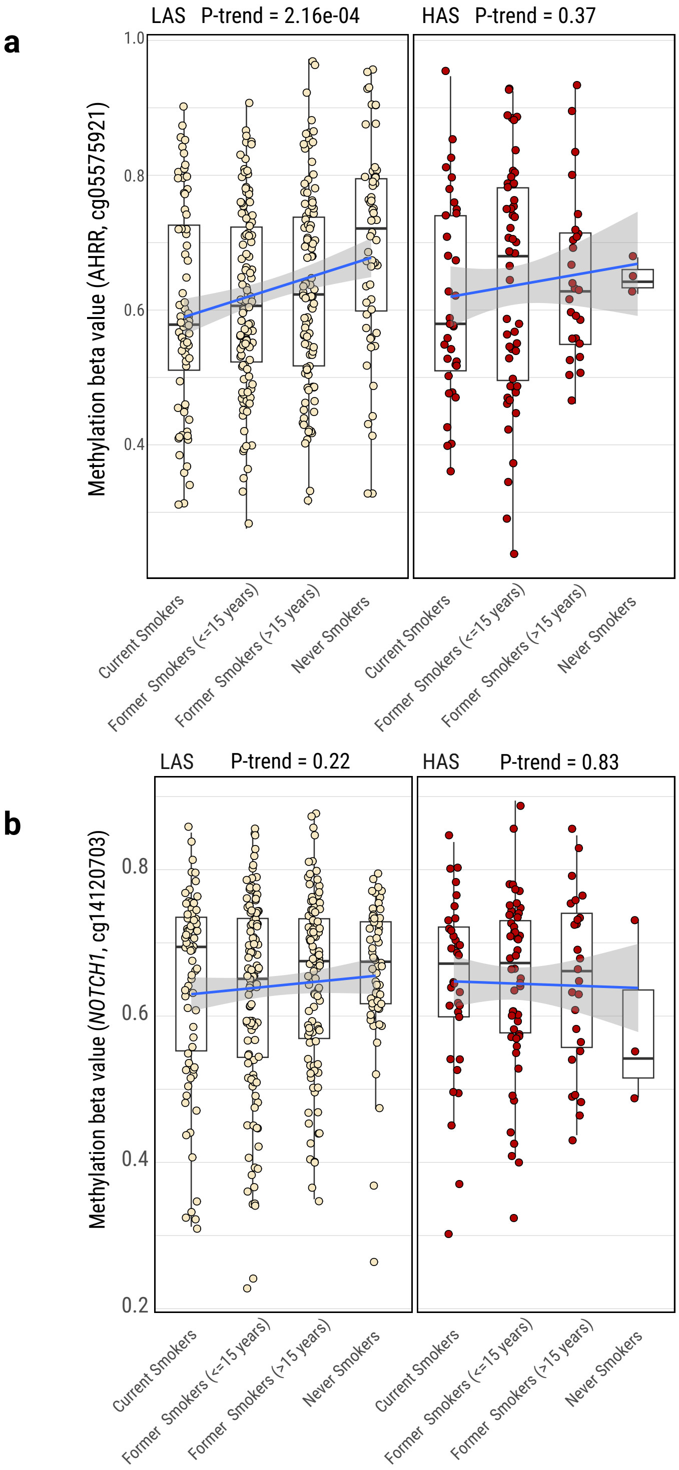

Supplementary Fig. 21: Association between smoking status and DNA methylation levels of the *AHRR* CpG probe cg05575921 and cg14120703 from *NOTCH1* in the TCGA LUAD dataset. The linear regression analyses are performed separately between LAS and HAS tumor. Trend test P-values (P_{trend}) are labeled above each subplot. All box plots display the median (centerline), interquartile range (box), and whiskers extending to 1.5× the interquartile range (IQR) by default in ggplot2. The shaded area represents the 95% confidence level.

Supplementary Fig. 22

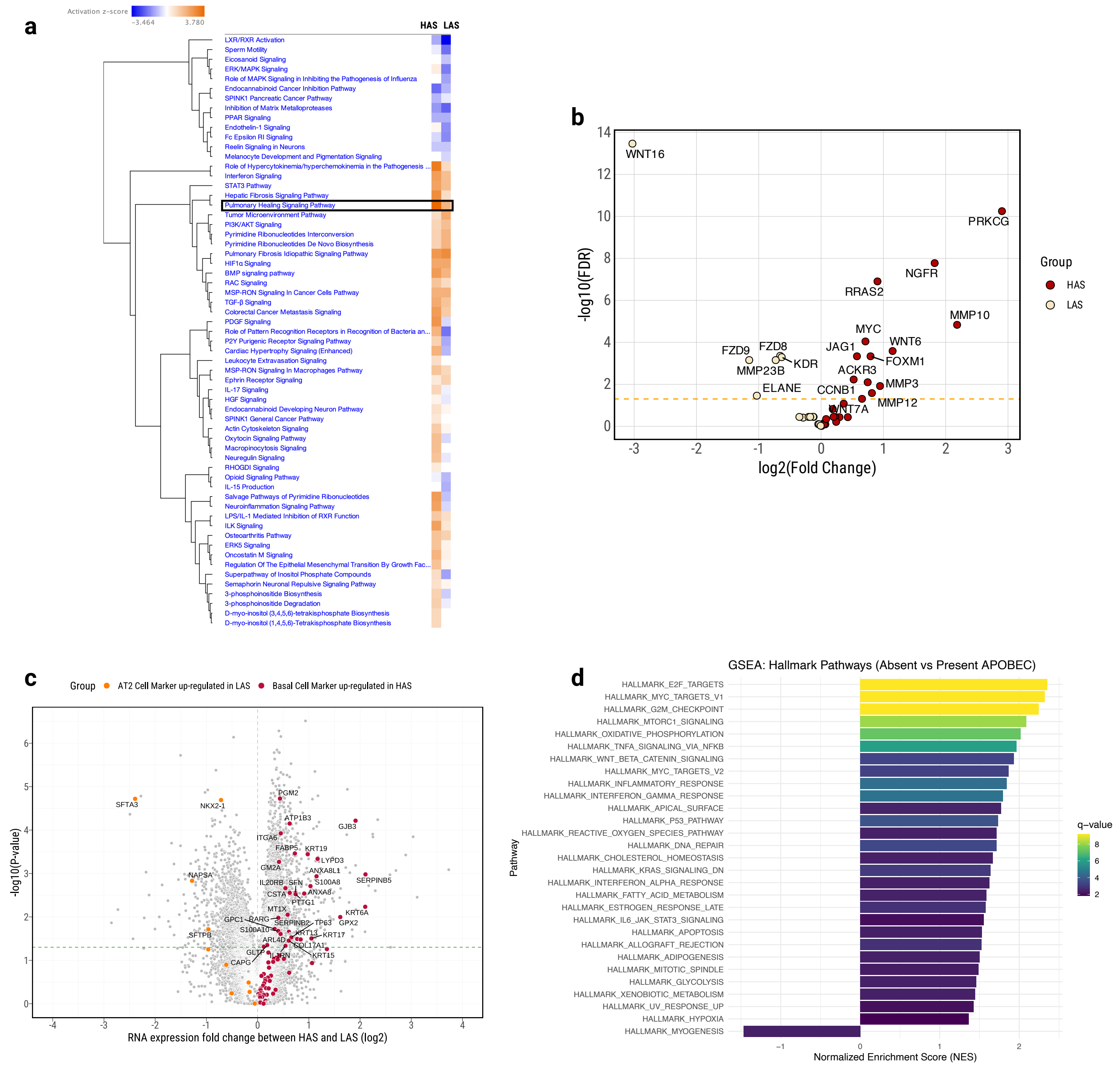

Supplementary Fig. 22: **Pulmonary healing signaling pathway and differential expression analysis between LAS and HAS tumors. a**, Clustering of pathways significantly associated with short TTFC in LUAD tumors from EAGLE (n=105). Pathway analyses using RNA-Seq data from lung tumor tissue separately between LAS and HAS tumors. Only pathways with absolute Z-score>1 and FDR<0.05 are included. **b**, Differential expression analysis between LAS and HAS tumors for genes involved in the pulmonary healing signaling pathway and significantly associated with TTFC. **c**, Volcano plot illustrating the differential expression analysis between LAS and HAS tumors for all protein-coding genes. Significantly upregulated AT2 cell markers in LAS tumors and basal cell markers in HAS tumors are labeled on the plot. The green line indicates the significance threshold ($P < 0.05$). **d**) Gene Set Enrichment Analysis (GSEA) using Hallmark gene sets (MSigDB, category "H") to compare transcriptomic profiles between HAS and LAS tumors.

Supplementary Fig. 23

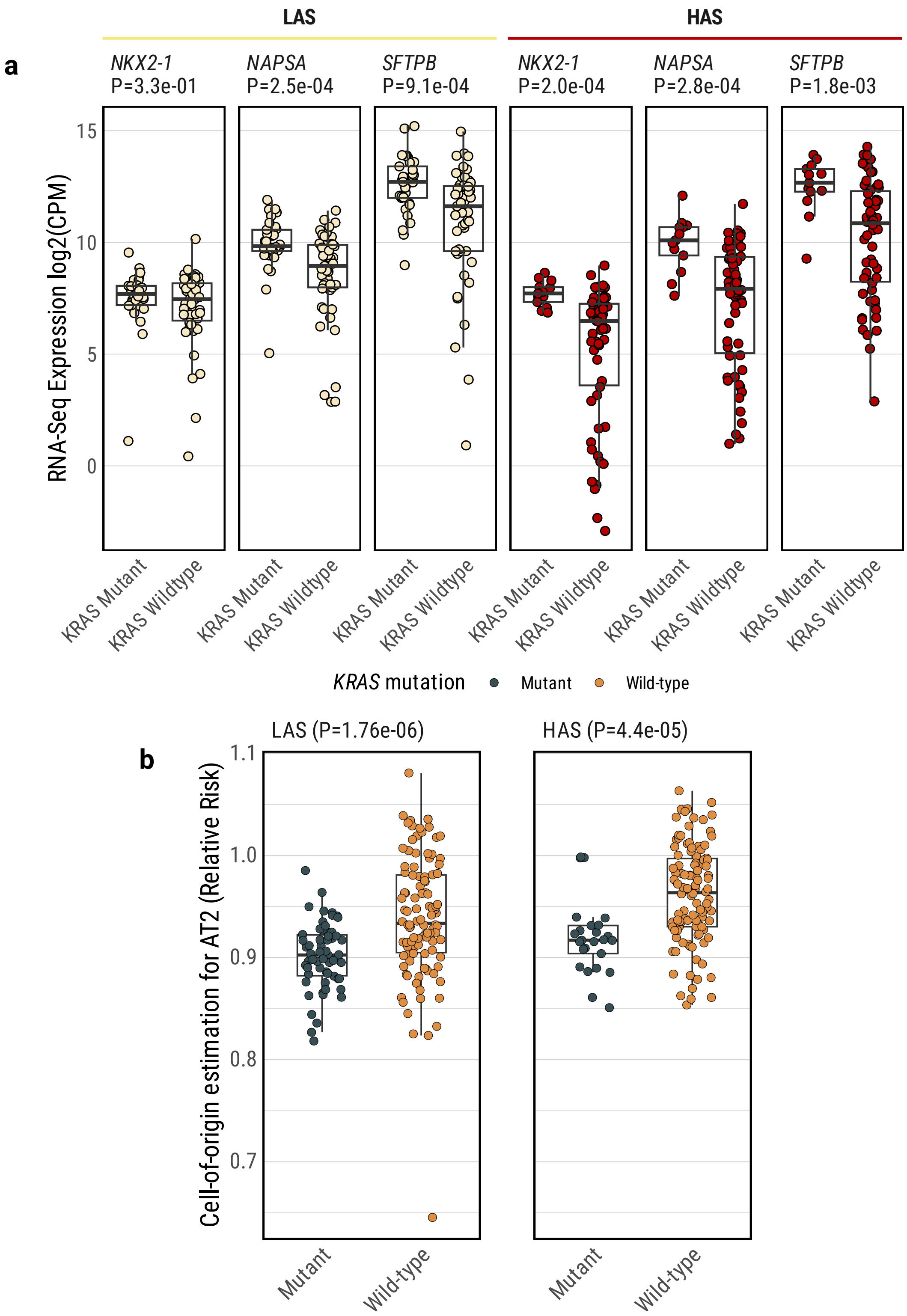

Supplementary Fig. 23: Differential expression of lung-specific cell type markers and cell of origin between *KRAS* wildtype and mutant tumors. a, Boxplots show the differentially expressed gene markers of lung-specific cell types between *KRAS* wildtype and mutant tumors, stratified by LAS and HAS tumors. P-values derived from the two-sided Wilcoxon rank-sum test are shown above the plots. **b**, The comparison of the estimated relative risk score of the AT2 cell of origin between *KRAS* wildtype and mutant tumors, stratified by APOBEC subtypes. The lower relative risk in each tumor sample indicates the likelihood of the putative cell of origin for that tumor. P-values derived from the Wilcoxon rank-sum test are shown on the right of the plot. All box plots display the median (centerline), interquartile range (box), and whiskers extending to 1.5× the interquartile range (IQR) by default in ggplot2.

Supplementary Fig. 24

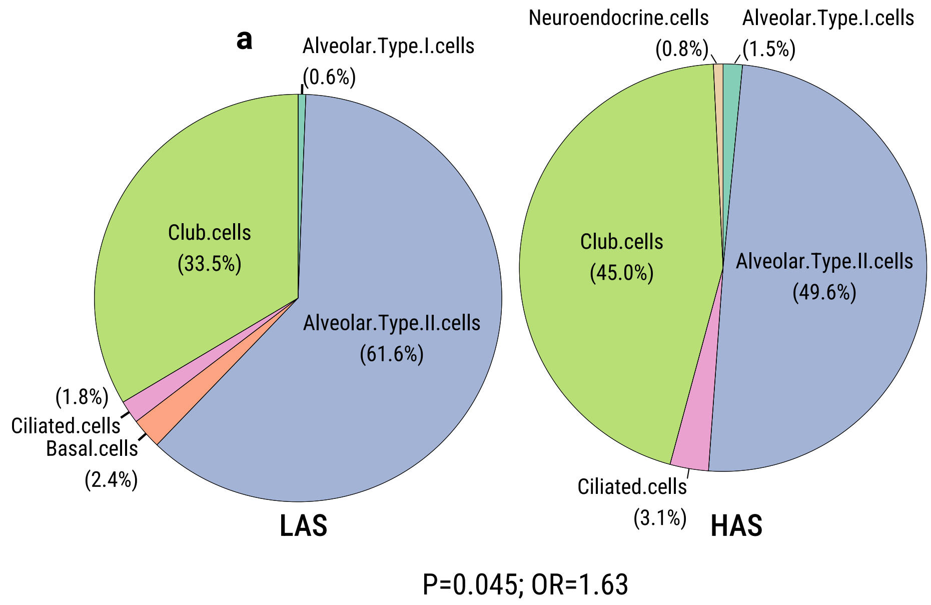

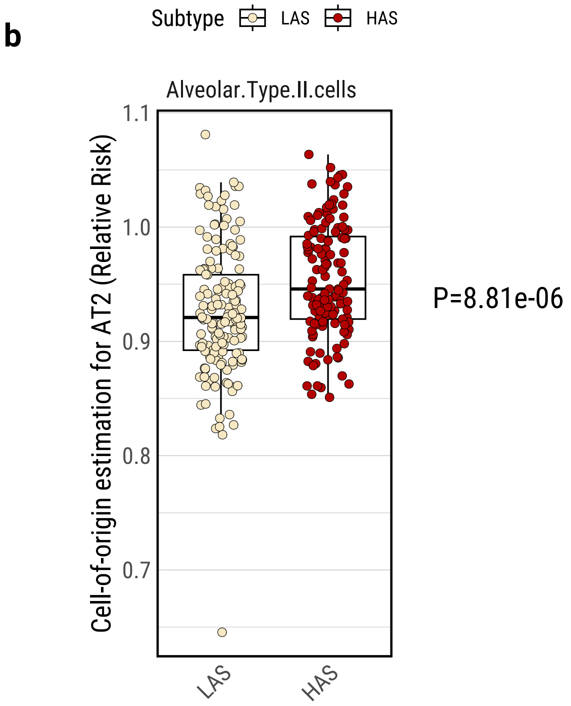

Supplementary Fig. 24: **Inferring patient-specific LUAD cell-of-origin from somatic mutational patterns. a,** Pie charts show the percentage of each inferred cell- of-origin in LAS and HAS tumors. P-values derived from the Fisher's exact test are shown on the bottom of the plot. **b**, The comparison of the estimated relative risk score of the AT2 cell of origin between LAS and HAS tumors. P-values derived from the two-sided Wilcoxon rank-sum test are shown on the right of the plot. Box plots display the median (centerline), interquartile range (box), and whiskers extending to 1.5× the interquartile range (IQR) by default in ggplot2.

Supplementary Fig. 25

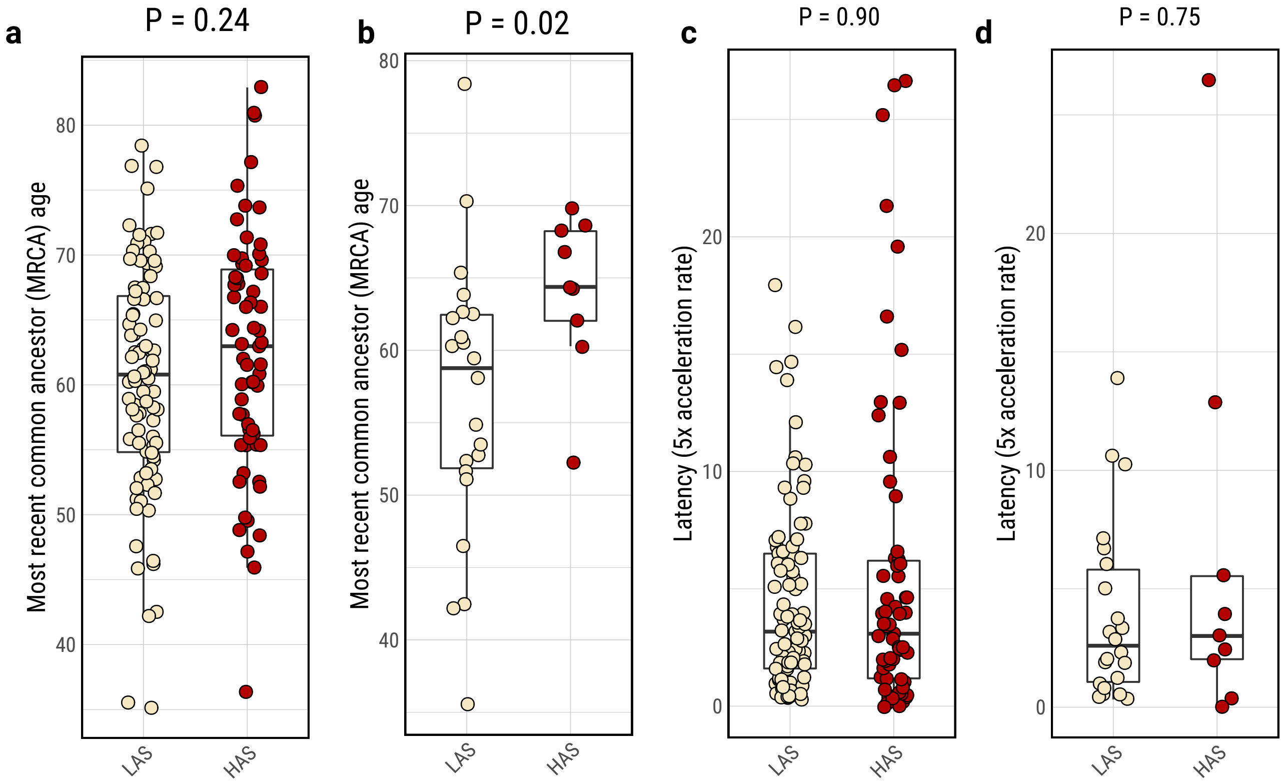

Supplementary Fig. 25: Comparison of the estimated age at the occurrence of the most recent common ancestor (MRCA) in all tumors (a) and in tumors with TTFC<=5 mins (b), and latency in all tumors (c) and in tumors with TTFC <=5 mins (d) between LAS and HAS tumors. Tumor cell latency is calculated as the difference between age at diagnosis and the estimated age at the occurrence of MRCA based on 5x acceleration rate. The P-value from the Wilcoxon sum rank test is shown above the boxplot. All box plots display the median (centerline), interquartile range (box), and whiskers extending to 1.5× the interquartile range (IQR) by default in ggplot2.

Supplementary Fig. 26

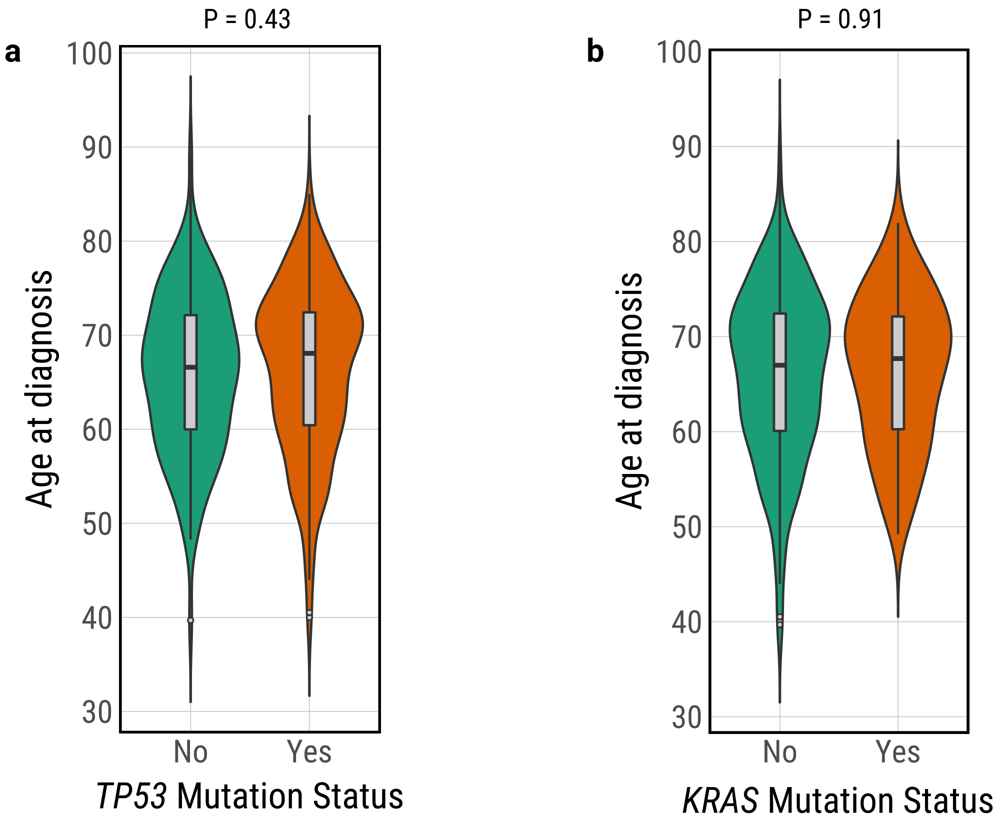

Supplementary Fig. 26: Age at diagnosis differences in patients based on *TP53* mutation (a) status and *RAS* mutation status (b). All box plots display the median (centerline), interquartile range (box), and whiskers extending to 1.5× the interquartile range (IQR) by default in ggplot2.

Supplementary Fig. 27

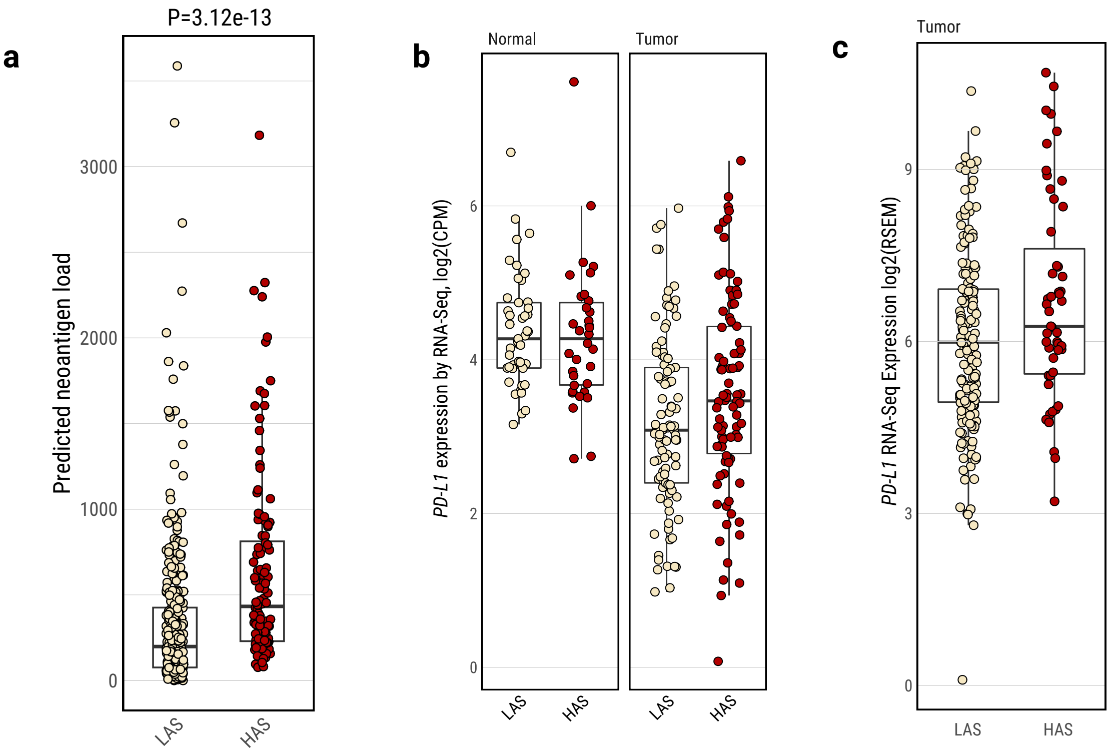

Supplementary Fig. 27: Immune-related features in LAS and HAS tumors. a, Comparison of neoantigen burden between LAS and HAS tumors from the TCGA LUAD dataset. P-values from two-sided Wilcoxon rank-sum tests are indicated above the boxplot. (**b-c**) PD-L1 expression differences between LAS and HAS tumors in (**b**) this study and (**c**) the TCGA LUAD study. All box plots display the median (centerline), interquartile range (box), and whiskers extending to 1.5× the interquartile range (IQR) by default in ggplot2.

Supplementary Fig. 28

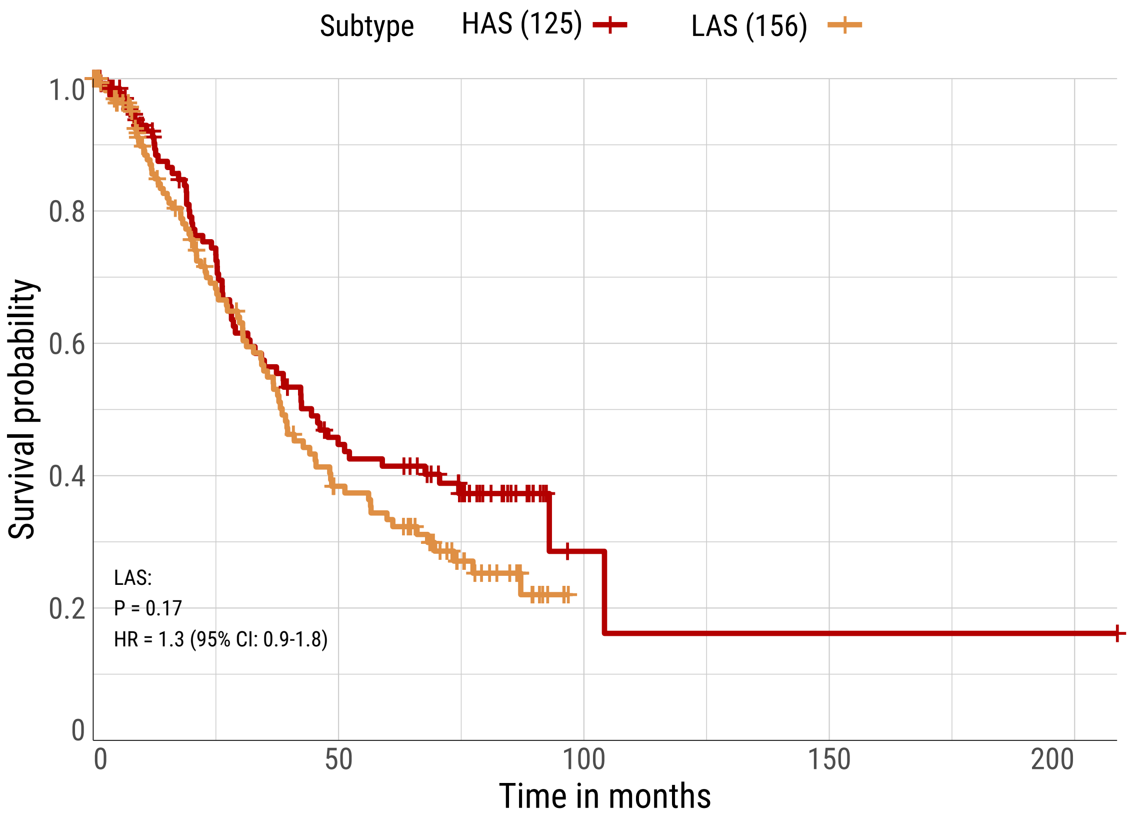

Supplementary Fig. 28: Kaplan–Meier survival curves for overall survival stratified by tumor subtype (LAS vs. HAS).

Supplementary Fig. 29

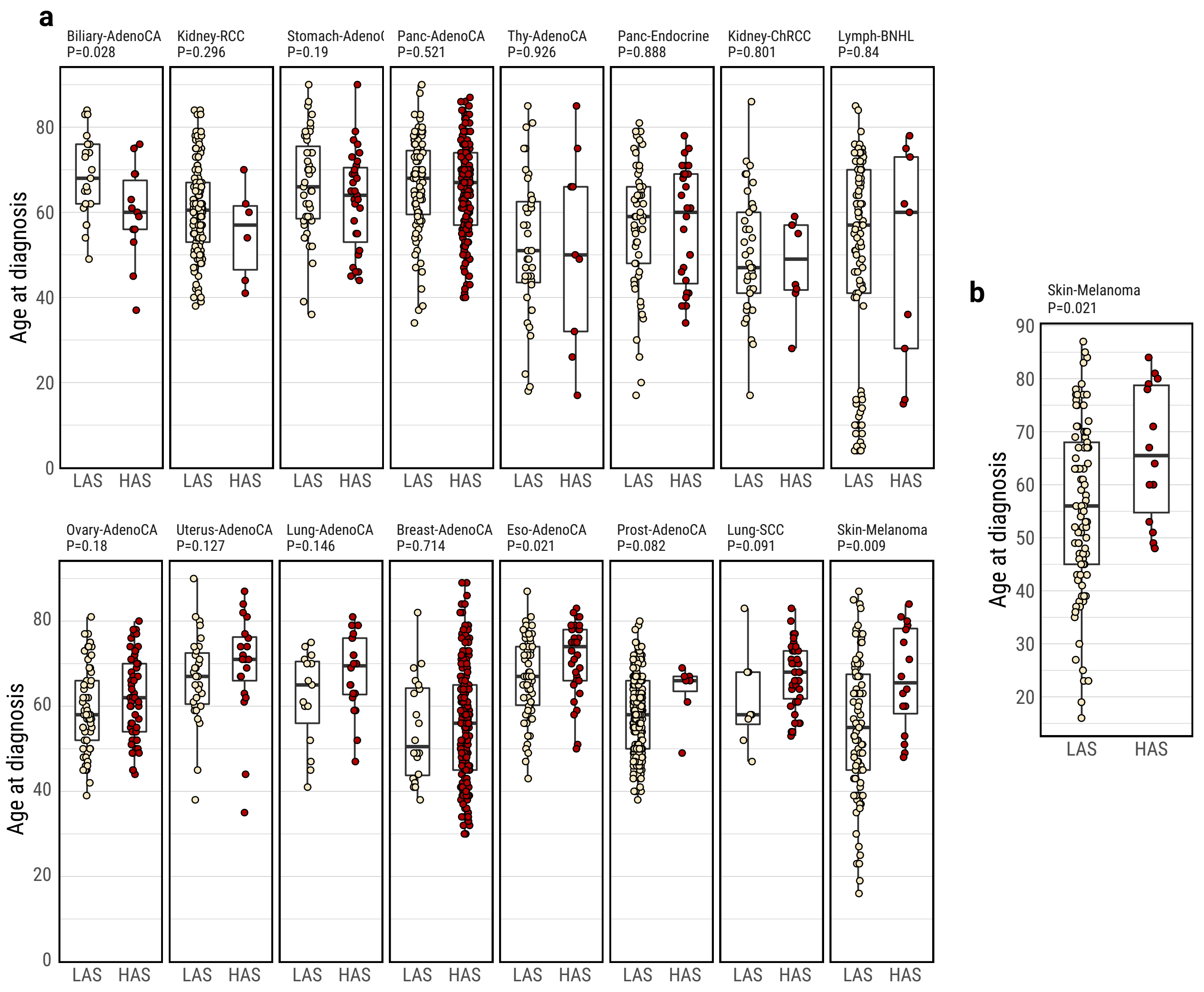

Supplementary Fig. 29: Comparison of age at diagnosis between LAS and HAS tumors from the PCAWG study across multiple cancer types using SBS2 and SBS13 to define HAS and LAS subtypes (a) and skin-melanoma only using SBS13 to define LAS and HAS subtypes (b). Only cancer types with sufficient tumors in both APOBEC LAS and HAS subtypes for comparison are included. P-values from the Wilcoxon sum rank test are shown above the boxplot. All box plots display the median (centerline), interquartile range (box), and whiskers extending to 1.5× the interquartile range (IQR) by default in ggplot2.

Supplementary Fig. 30

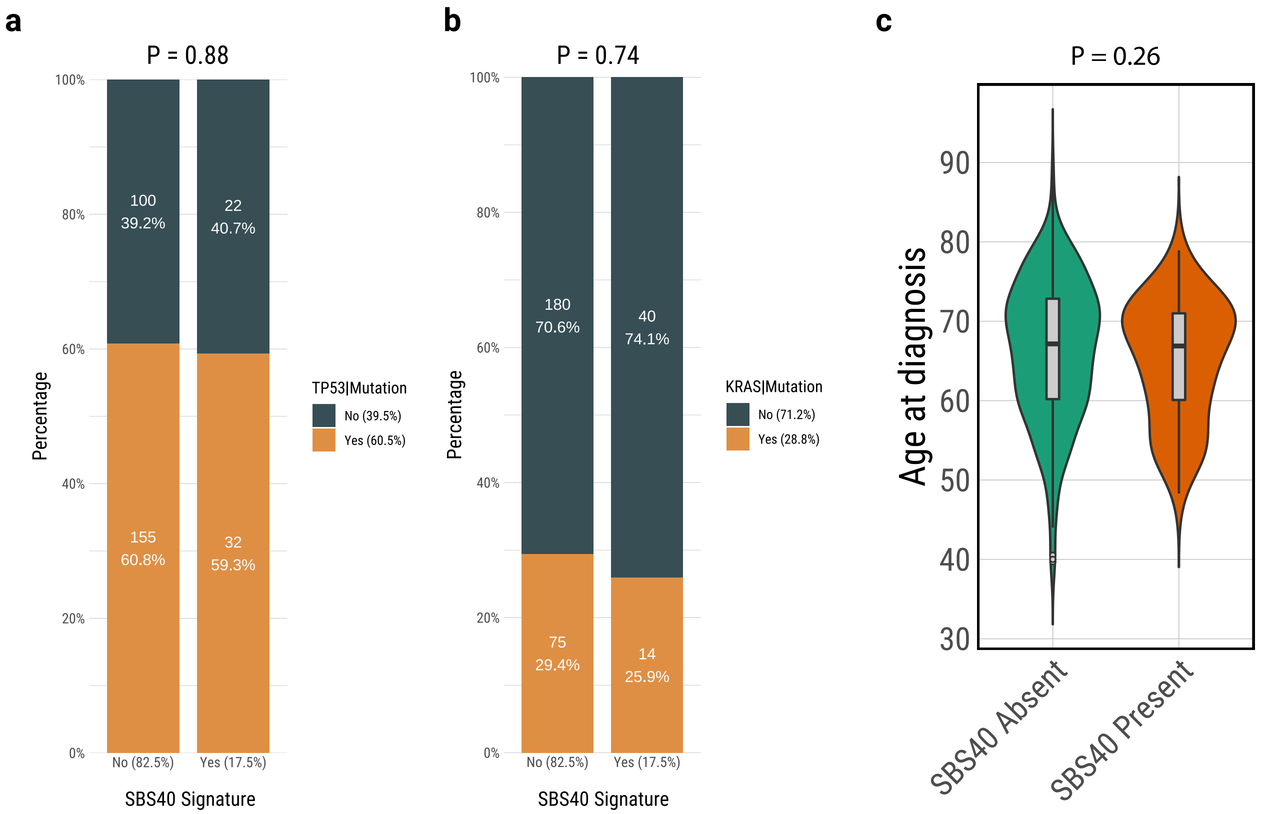

Supplementary Fig. 30: Comparisons of tumors stratified based on the detection of SBS40 signatures with regard to *TP53* mutation frequency (a), *KRAS* mutation frequency (b) and age at diagnosis (c). P-values of the Fisher's exact test or two-sided Wilcoxon sum rank test are shown on the top of each bar plot. Box plots display the median (centerline), interquartile range (box), and whiskers extending to 1.5× the interquartile range (IQR) by default in ggplot2.

Extended Data Fig. 31

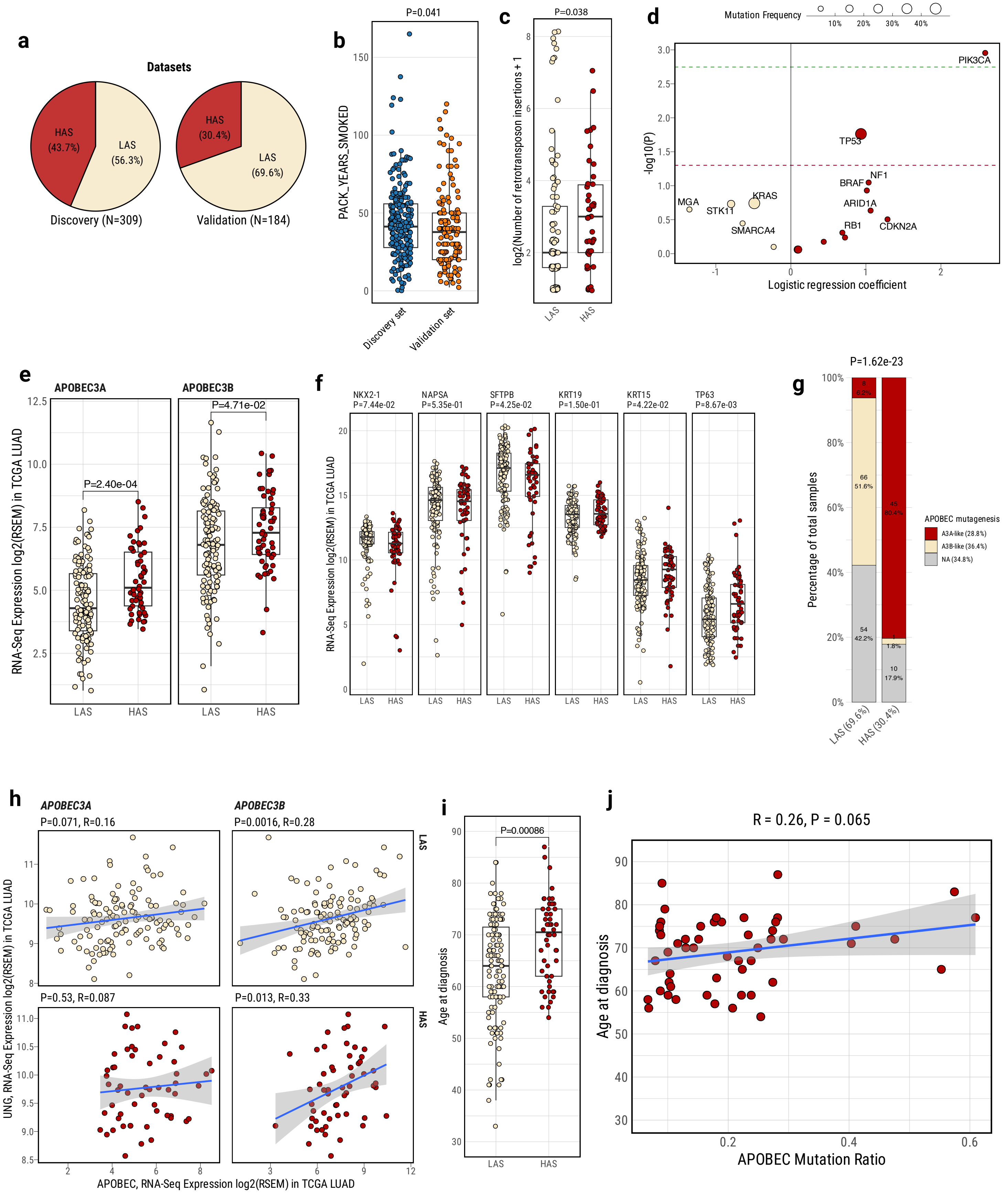

Supplementary Fig. 31: Validation of APOBEC influencing age at tumor onset in an independent TCGA LUAD WGS dataset. a, Proportion of LAS and HAS tumors in both the discovery and validation datasets. **b**, Comparison of smoking exposure, measured in pack-years, between patients in the discovery and validation datasets. **c**, Number of retrotransposon insertions between LAS and HAS tumors. **d**, Logistic regression analysis of tumor subtypes and nonsynonymous mutation status of driver genes, adjusting for covariates including age, sex, histology, TMB, and tumor purity. Significance thresholds are indicated with dashed lines for $P<0.05$ (red) and $FDR<0.05$ (green). **e**, Differential expression of APOBEC family genes between LAS and HAS tumor samples. P-values from Wilcoxon rank-sum tests are labeled above the boxplot. **f**, Boxplots showing differentially expressed gene markers of lung-specific cell types between LAS and HAS LUAD tumors (n=155). **g**, Proportions of A3A-like and A3B-like mutagenesis between LAS and HAS tumors. Tumors not enriched with TCA mutations or without significant differences between RTCA and YTCA mutations are classified as N/A. **h**, Gene expression correlation between UNG and *APOBEC3A* (left) or *APOBEC3B* (right), stratified by LAS (top) and HAS (bottom) tumors. Significant P-values and Pearson correlation coefficients are shown above each scatter plot. **i**, Difference in age at diagnosis between LAS and HAS tumors. **j** Correlation between APOBEC mutation ratio and age at diagnosis in HAS tumors. All box plots display the median (centerline), interquartile range (box), and whiskers extending to 1.5× the interquartile range (IQR) by default in ggplot2. The shaded area represents the 95% confidence level.
