# Supplementary material for: APOBEC affects tumor evolution and age at onset of lung cancer in smokers
Source: Nat Commun. 2025 May 21;16:4711. doi: 10.1038/s41467-025-59923-8 (PMC12092836; doi:10.1038/s41467-025-59923-8)
Supplement: Supplementary file 4 — Description of Additional Supplementary Files [file 41467_2025_59923_MOESM4_ESM.pdf]

Description of Additional Supplementary Files

Supplementary Data 1
Description: Overall data summary and genomic features included in this study.

Supplementary Data 2
Description: Mutational signature deconvolution for SBS, DBS, and ID mutation types using the COSMIC mutational signature catalog as reference.

Supplementary Data 3
Description: Smoking history information for the EAGLE study subjects.

Supplementary Data 4
Description: Distinct patterns of mutagenesis by APOBEC cytidine deaminases using the P-MACD algorithm. Enrichment analyses were performed using two-sided Fisher's exact tests, with multiple testing correction applied using the Benjamini–Hochberg (BH) method, as implemented in the P-MACD algorithm.

Supplementary Data 5
Description: RNA-Seq expression data quantified as log2CPM for genes involved in the major analyses.

Supplementary Data 6
Description: Methylation beta value for 952 CpG probes that were found to have genome-wide significant associations with smoking variables in a previous study[59].

Supplementary Data 7
Description: Associations between smoking variables and methylation levels of known smoking-related CpG probes in both normal and tumor tissue samples. Multivariate association analyses were performed using linear regression, adjusting for age, sex, histology, and tumor purity. Multiple testing correction was applied using the Benjamini–Hochberg (BH) method.

Supplementary Data 8
Description: Differentially expressed gene markers of lung-specific cell types between LAS and HAS LUAD tumors (n=155). Two statistical methods were included: linear regression with adjustment for copy number alterations and tumor purity, and two-sided Mann–Whitney U test.

Supplementary Data 9
Description: The APOBEC subtype information from the new TCGA LUAD WGS dataset included as validation dataset.
